# Multiomics analyses reveal high yield-related genes in the hypothalamic-pituitary-ovarian/liver axis of chicken

**DOI:** 10.1016/j.psj.2024.104276

**Published:** 2024-09-02

**Authors:** Jun’an Shi, Hanlin Xiong, Junchao Su, Qigui Wang, Haiwei Wang, Chaowu Yang, Chenming Hu, Zhifu Cui, Lingbin Liu

**Affiliations:** ⁎ College of Animal Science and Technology, Chongqing Key Laboratory of Herbivore Science, Southwest University, Beibei, 400700, Chongqing, China; † ChongQing Academy of Animal Sciences, Rongchang, Chongqing 402460, China; ‡ Sichuan Animal Science Academy, Animal Breeding and Genetics Key Laboratory of Sichuan Province, Chengdu 610066, China

**Keywords:** hypothalamic-pituitary axis, liver-ovarian axis, transcriptome, metabolome, egg production

## Abstract

Egg production, regulated by multiple tissues, is among the most important economic traits in poultry. However, current research only focuses on the hypothalamic-pituitary-ovarian axis, ignoring the most important organ for substance metabolism in the body, the liver. Eggs are rich in lipids, proteins, and other nutrients, which are biosynthesized in the liver. Therefore, here the liver was included in the study of the hypothalamic-pituitary axis. This study used hypothalamus (HH_vs_LH), pituitary (HP_vs_LP), liver (HL_vs_LL), and ovary (HO_vs_LO) tissue samples from high- and low-laying Chengkou mountain chickens (**CMC**) for epihistological, transcriptome and metabolomic analyses aimed at improving the reproductive performance of CMC. The results showed that the liver of the high-laying group was yellowish, the cell boundary was clear, and the lipid droplets were evenly distributed. The ovaries of the high-laying group had a complete sequence of hierarchical follicles, which were rich in yolk. In contrast, the ovaries of the low-laying group were atrophic, except for a few small yellow follicles, and numerous primordial follicles that remained. The transcriptome sequences yielded 167.11 Gb of clean data, containing 28,715 genes. Furthermore, 285, 822, 787, and 1,183 differentially expressed genes (**DEG**) were identified in HH_vs_LH, HP_vs_LP, HL_vs_LL and HO_vs_LO and the DEGs significantly enriched 77, 163, 170, 171 pathways, respectively. Metabolome sequencing yielded 21,808 peaks containing 4,006 metabolites. The differential metabolite analysis yielded 343 and 682 significantly different metabolites (**SDM**) that significantly enriched 136 and 87 pathways in the liver and ovaries, respectively. A combined analysis of the transcriptome and metabolome of the liver and ovaries identified “*CYP51A1*-4α-carboxy-stigmasta7, 24(24(1))-dien-3β-ol” and “*ACSS1B*-estrone 3-sulfate” and other multiple gene-metabolite pairs. The DEGs in the hypothalamus and pituitary mainly enriched signaling transduction. In contrast, the DEGs and SDMs in the liver and ovaries mainly enriched the substance metabolism pathways: “gap junction”, “extracellular matrix (ECM)-receptor interaction”, “Steroid biosynthesis”, and “Steroid hormone biosynthesis”. These results suggest that the hypothalamic-pituitary axis may affect egg production mainly by regulating lipid metabolism in the liver and ovaries.

## INTRODUCTION

Eggs are among the most important sources of animal protein, widely loved by the public because of their rich and balanced nutrients, easy absorption, and affordable price (Réhault-Godbert et al., 2019). For fitness enthusiasts, eggs are an indispensable food. After many years of cultivation, the laying of chickens has dramatically improved and the global annual production of commercial laying chicken lines exceeds 300 eggs, thanks to continual cultivation. The laying rate of female birds is the most important reproductive trait, mainly regulated by the hypothalamic-pituitary-gonadal axis and other endocrine organs and tissues (Li et al., 2015, Bornelöv et al., 2018, Zhang et al., 2018). Moreover, the liver, a major organ for carbohydrate, lipid, and protein metabolism, energy regulation, and hormone production, influences egg production traits through various biological processes (Réhault-Godbert and Guyot, 2018, Nimisha et al., 2022, Wang, 2023).

The Chengkou Mountain Chicken (**CMC**) is an outstanding local chicken breed in China, with the distinctive characteristics of a local, high-quality breed, including resistance to coarse feeding, delicious meat, strong disease resistance, and high nutritional value (Shi et al., 2022, Liu et al., 2022b), but with poor reproductive performance. Investigating the complex regulatory mechanisms of egg laying helps improve egg quality and production, promotes the genetic improvement of local chicken breeds, and increases economic efficiency.

In poultry breeding, transcriptomics has been widely used due to the improved sequencing depth and decreased sequencing cost (Li et al., 2015, Nimisha et al., 2022, Mantri et al., 2021, Chen et al., 2022, Li et al., 2022b, Pham et al., 2022). Meanwhile, metabolomics is an important tool in the postgenome era because post-transcriptionally regulated small-molecule metabolites are the end products of biochemical processes (X et al., 2023). Therefore, metabolomics can be used to investigate the physiological reasons underlying egg production by identifying small-molecule metabolites. Previously, metabolomics had been used to study poultry reproduction (Nimisha et al., 2022), gut function (Rzeznitzeck et al., 2022), immunity (Kuang et al., 2020), and meat quality (Tan et al., 2021, Zhang et al., 2021). Therefore, metabolomics is better suited for analysis with other omics technologies, such as transcriptomics, to reveal biological processes and combined metabolomics and transcriptomics analyses have been employed to study chicken quality (Li et al., 2022a, Gai et al., 2023), microbiome immunity (Shen et al., 2022), and meat quality (Liu et al., 2023). Also, metabolomics and proteomics were employed to analyze the liver and ovarian differences in chickens (Tang et al., 2023, Cui et al., 2022), along with genome wide association study analysis of the serum metabolic profiles of chickens (Tian et al., 2023).

Although many studies have been conducted, there is limited knowledge of the effects of the hypothalamic-pituitary-ovarian axis and liver on egg production in poultry. Thus, this study examined the hypothalamic-pituitary system and sequenced the transcriptome and metabolome of the ovaries and liver of CMC to reveal the transcriptional regulatory mechanisms affecting egg-laying performance in CMC.

## RESULTS

### Morphological and Histological Characteristics of the Liver and Ovaries of High- and Low-Laying Chickens

**Figure 1:**
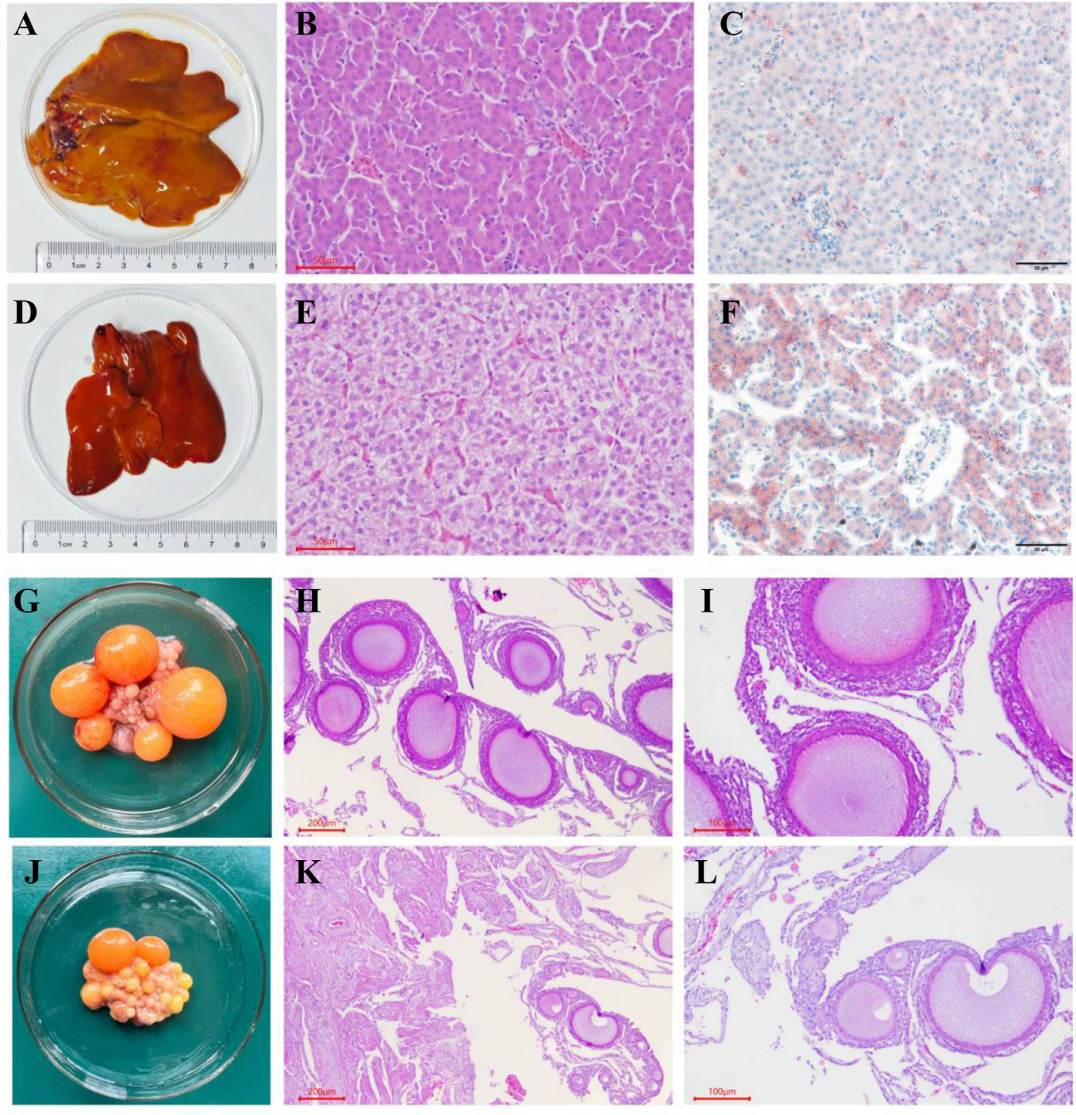
Ovarian morphological and histological characteristics of high-laying- and low-laying-chickens. (A) The liver from the high-laying group; (B, C). Hematoxylin-eosin staining (H&E) and Oil Red O results of the liver of the high-laying group; (D) The liver of the low-laying group; (E, F). H&E and Oil Red O of the liver of the low-laying group; (G) The ovary of the high-laying group (H, I). H&E of the ovary of the high-laying group (H 40 ×, I 100 ×); (J). The ovary of the low-laying group; (K, L). H&E of the ovary of the low-laying group (K 40 ×, L 100 ×).

The liver color of the high-laying group was lighter than that of the low-laying group (Figures 1A and 1D). In the high-laying group, Hematoxylin–Eosin (**H&E**) staining showed that the cytoplasm of the hepatocytes was mildly stained, the nuclei of the cells were darkly stained, and the cell boundaries were clear (Figure 1B). Oil Red O staining showed a narrow cellular interstitial space and the lipid droplets were uniformly distributed (Figure 1C). In contrast, the hepatocytes of the low-laying group were littery and unclearly arranged, with a light pink cytoplasm and light blue nuclei (Figure 1E). Oil Red O staining also revealed large lipid droplets, with large gaps between the lipid droplets, and the cells were nonuniformly arranged (Figure 1F). Furthermore, the complete sequence of hierarchical follicles was filled with large amounts of egg yolk deposits in the ovaries of the high-laying group, but only a few small yellow follicles in the ovaries of the low-laying group (Figures 1G and J). Then, H&E staining showed numerous graded follicles in the ovaries of the high-laying group (Figure 1H) with a dense layer of granulosa cells (Figure 1I). In contrast, numerous primordial follicles and a sparse granulosa cell layer were observed in the ovaries of the low-laying group (Figures 1K and L).

### Transcriptome Analysis of High- and Low-Laying Chicken Groups

**Figure 2:**
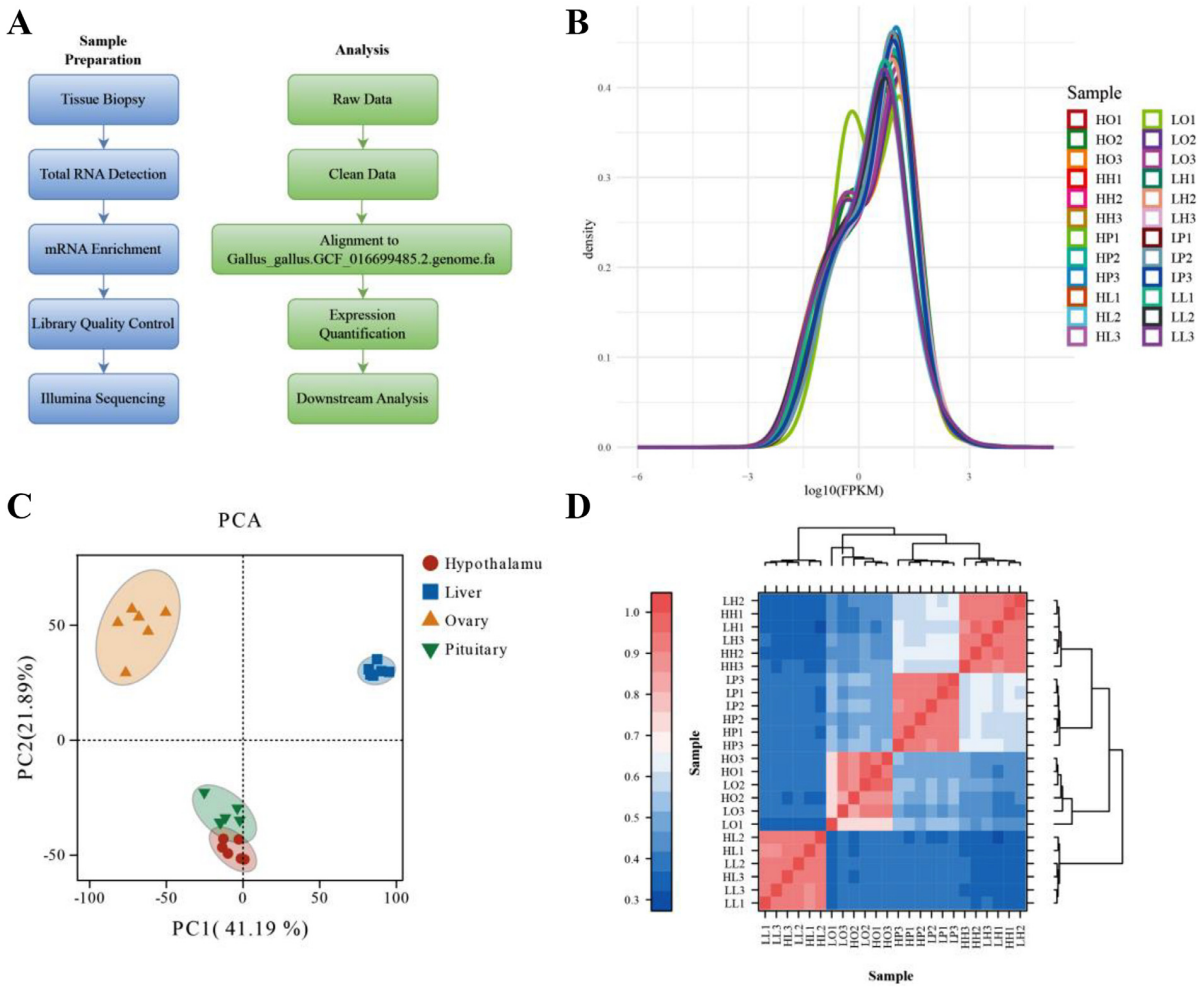
Methodology and transcriptome data overview. (A) A flowchart of sample preparation (blue) and analytical methods (green) were used in this study. (B) FPKM distribution of mRNA. (C) Principal component analysis (PCA) distribution of all samples. (D) Heatmap of sample correlation.

Figure 2A shows an overview of the transcriptome sample preparation and analytical approaches used in this study. The hypothalamus, pituitary, ovaries, and liver were obtained from 6 chickens to construct 24 cDNA libraries. After quality control, 167.11 Gb of clean data was obtained, and the percentage of Q30 bases in each sample exceeded 93.99% (Table 1, see supplementary material). The reads mapped to the reference genome at 92.55 to 95.72% (Table 2, see the supplement material), with 80% mapping to the exon region and a 1:1 intron:intergene ratio (Supplementary Figure S1). Overall, the mapped reads contained 28,715 genes, including 10,922 novel ones.

**Table 1:** Sequencing data statistics.

Samples	Clean reads	Clean bases	GC Content	%≥Q30
HO1	26,652,416	7,970,338,194	47.96%	94.98%
HO2	25,859,859	7,729,736,704	47.30%	94.81%
HO3	24,243,337	7,251,648,216	48.14%	94.61%
HH1	22,823,055	6,820,172,214	47.20%	94.48%
HH2	21,289,563	6,364,065,062	47.07%	94.57%
HH3	21,294,861	6,364,435,582	46.95%	94.64%
HP1	22,062,298	6,591,474,458	47.32%	94.41%
HP2	21,544,251	6,436,197,114	47.54%	94.49%
HP3	20,062,957	5,995,654,866	47.52%	94.31%
HL1	25,514,315	7,636,628,126	46.98%	95.09%
HL2	23,961,158	7,172,533,754	47.06%	94.95%
HL3	25,589,486	7,657,992,234	47.85%	95.27%
LO1	24,349,647	7,281,486,640	47.18%	94.78%
LO2	24,374,853	7,288,102,454	48.48%	93.99%
LO3	25,755,987	7,703,836,662	47.88%	94.52%
LH1	24,005,567	7,176,534,980	46.96%	94.63%
LH2	19,926,723	5,956,343,590	47.02%	94.38%
LH3	22,467,631	6,715,023,816	46.72%	94.23%
LP1	19,372,177	5,786,128,294	48.39%	94.11%
LP2	21,192,209	6,330,157,450	48.98%	94.06%
LP3	21,673,777	6,475,528,866	48.86%	94.26%
LL1	21,766,434	6,513,440,156	48.03%	94.92%
LL2	28,313,602	8,473,360,832	46.98%	94.79%
LL3	24,787,143	7,418,304,240	47.52%	95.03%

Abbreviations: HO/LO, high-laying chickens’ ovary/low-laying chickens’ ovary; HH/LH: high-laying chickens’ hypothalamus/low-laying chickens’ hypothalamus; HP/LP, high-laying chickens’ pituitary/low-laying chickens’ pituitary; HL/LL, high-laying chickens’ liver/low-laying chickens’ liver. The same as below.

**Table 2:** Statistical of alignment results between sequencing data and reference genomes.

Sample	Total reads	Mapped reads	Unique mapped reads	Multiple map reads	Reads map to "+"	Reads map to "-"
HO1	53,304,832	50,464,214 (94.67%)	49,653,163 (93.15%)	811,051 (1.52%)	25,752,119 (48.31%)	25,751,161 (48.31%)
HO2	51,719,718	48,730,052 (94.22%)	47,886,260 (92.59%)	843,792 (1.63%)	24,937,401 (48.22%)	24,910,806 (48.17%)
HO3	48,486,674	45,835,625 (94.53%)	45,046,340 (92.90%)	789,285 (1.63%)	23,422,960 (48.31%)	23,429,943 (48.32%)
HH1	45,646,110	42,714,531 (93.58%)	42,227,364 (92.51%)	487,167 (1.07%)	21,689,523 (47.52%)	21,667,057 (47.47%)
HH2	42,579,126	40,052,490 (94.07%)	39,538,240 (92.86%)	514,250 (1.21%)	20,375,235 (47.85%)	20,347,979 (47.79%)
HH3	42,589,722	40,120,833 (94.20%)	39,671,443 (93.15%)	449,390 (1.06%)	20,353,495 (47.79%)	20,346,580 (47.77%)
HP1	44,124,596	41,142,579 (93.24%)	40,263,028 (91.25%)	879,551 (1.99%)	21,251,707 (48.16%)	21,089,850 (47.80%)
HP2	43,088,502	40,227,958 (93.36%)	39,081,917 (90.70%)	1,146,041 (2.66%)	20,954,981 (48.63%)	20,828,674 (48.34%)
HP3	40,125,914	37,779,145 (94.15%)	37,134,249 (92.54%)	644,896 (1.61%)	19,327,961 (48.17%)	19,287,120 (48.07%)
HL1	51,028,630	48,823,377 (95.68%)	47,403,731 (92.90%)	1,419,646 (2.78%)	25,334,860 (49.65%)	25,332,096 (49.64%)
HL2	47,922,316	45,871,808 (95.72%)	44,515,666 (92.89%)	1,356,142 (2.83%)	23,871,028 (49.81%)	23,859,505 (49.79%)
HL3	51,178,972	48,822,993 (95.40%)	47,503,476 (92.82%)	1,319,517 (2.58%)	25,267,002 (49.37%)	25,278,299 (49.39%)
LO1	48,699,294	45,724,561 (93.89%)	44,815,761 (92.03%)	908,800 (1.87%)	23,549,252 (48.36%)	23,559,464 (48.38%)
LO2	48,749,706	45,677,309 (93.70%)	44,844,666 (91.99%)	832,643 (1.71%)	23,371,645 (47.94%)	23,376,557 (47.95%)
LO3	51,511,974	48,717,358 (94.57%)	47,824,182 (92.84%)	893,176 (1.73%)	24,964,426 (48.46%)	24,913,036 (48.36%)
LH1	48,011,134	45,147,989 (94.04%)	44,617,097 (92.93%)	530,892 (1.11%)	22,932,443 (47.76%)	22,917,474 (47.73%)
LH2	39,853,446	37,455,389 (93.98%)	37,059,634 (92.99%)	395,755 (0.99%)	18,985,990 (47.64%)	18,977,910 (47.62%)
LH3	44,935,262	42,225,875 (93.97%)	41,721,577 (92.85%)	504,298 (1.12%)	21,450,649 (47.74%)	21,434,646 (47.70%)
LP1	38,744,354	36,079,181 (93.12%)	35,366,845 (91.28%)	712,336 (1.84%)	18,586,800 (47.97%)	18,541,997 (47.86%)
LP2	42,384,418	39,227,015 (92.55%)	38,615,789 (91.11%)	611,226 (1.44%)	20,011,181 (47.21%)	20,000,988 (47.19%)
LP3	43,347,554	40,481,857 (93.39%)	39,679,572 (91.54%)	802,285 (1.85%)	20,815,259 (48.02%)	20,763,143 (47.90%)
LL1	43,532,868	41,275,324 (94.81%)	40,312,216 (92.60%)	963,108 (2.21%)	21,267,217 (48.85%)	21,253,703 (48.82%)
LL2	56,627,204	54,022,085 (95.40%)	52,518,814 (92.74%)	1,503,271 (2.65%)	28,106,709 (49.63%)	27,978,601 (49.41%)
LL3	49,574,286	47,239,482 (95.29%)	45,868,348 (92.52%)	1,371,134 (2.77%)	24,522,256 (49.47%)	24,513,763 (49.45%)

Transcript expression was presented by the FPKM (Fragments per kilo-base of exon per million fragments mapped) value, as shown in Figure 2B. The 2 characteristic values of the horizontal and vertical coordinates reflected the complex sample composition relationship, revealing the “main” element and structure of the data. Moreover, it aided in exploring the distance relationship between samples. All samples were significantly divided into 4 sections according to the different tissues (Figure 2C), and the heat map also clustered the samples accordingly (Figure 2D). Altogether, the results were reliable and reproducible between samples, ensuring their veracity in the subsequent analysis.

**Figure 3:**
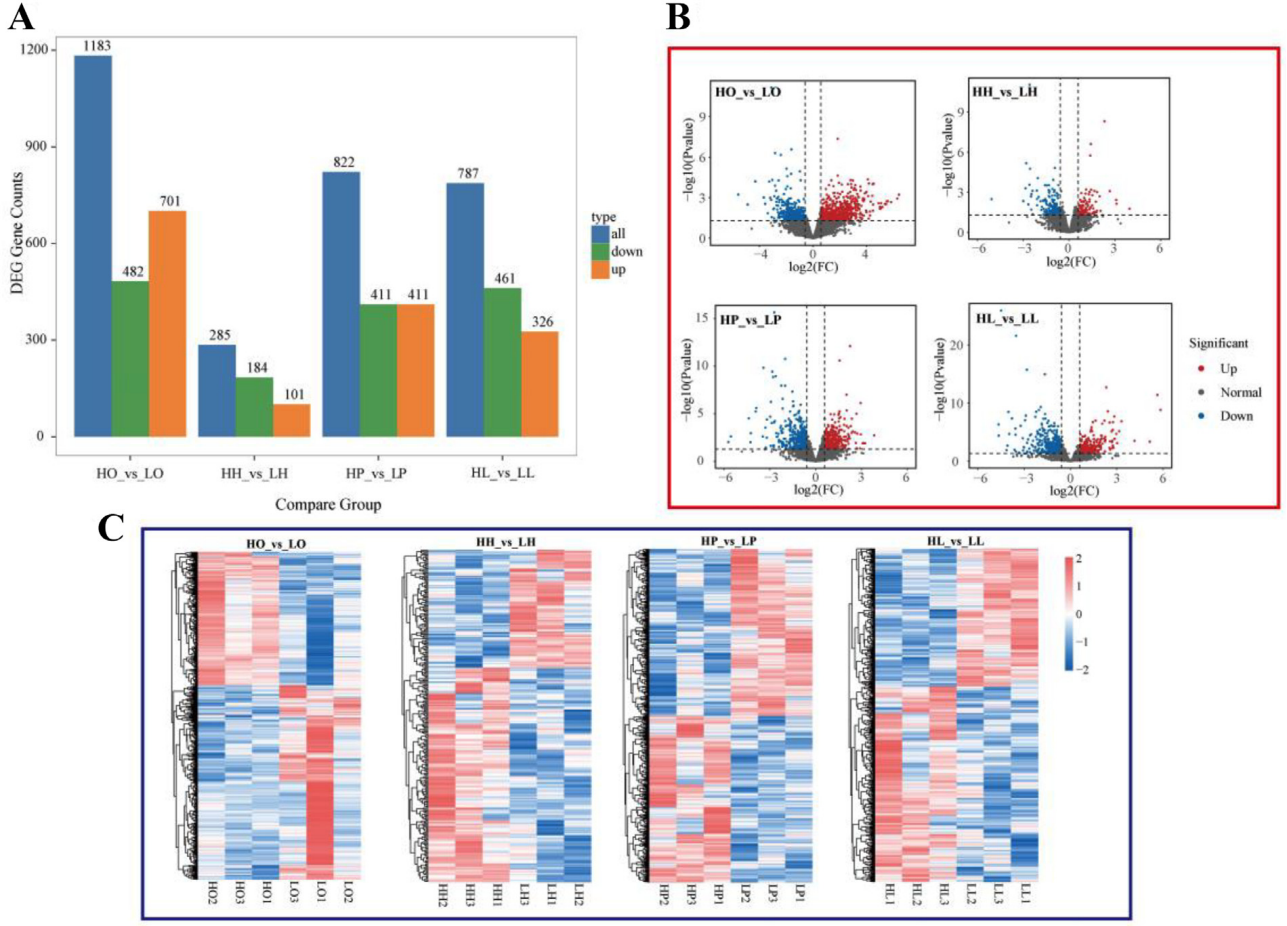
Differentially expressed genes (DEGs). (A) Number of DEG. (B) Volcano plots of DEG. (C) Heatmap of DEG. Up: the expression of group B was upregulated relative to group A; Down: the expression of group B was downregulated relative to group A.

The differential analysis of transcriptome data was performed using DESeq2. In total, 285, 822, 787, and 1,183 differentially expressed genes (DEGs) were identified in group HH_vs_LH, HP_vs_LP, HL_vs_LL, and HO_vs_LO, respectively (Figure 3A, Supplementary Table S1). Subsequently, to better demonstrate the expression of DEGs in the samples, a total gene expression volcano map (Figure 3B) and a DEGs expression heat map (Figure 3C) were drawn for each group. In the volcano map, blue dots represent genes that are significantly under-expressed in the high-laying group, and red dots represent genes that are significantly high-expressed in the high-laying group. In the heat map, high-expressed genes appear red and low-expressed genes appear blue. There is excellent repeatability between samples, proving the consistency of the sampling.

**Figure 4:**
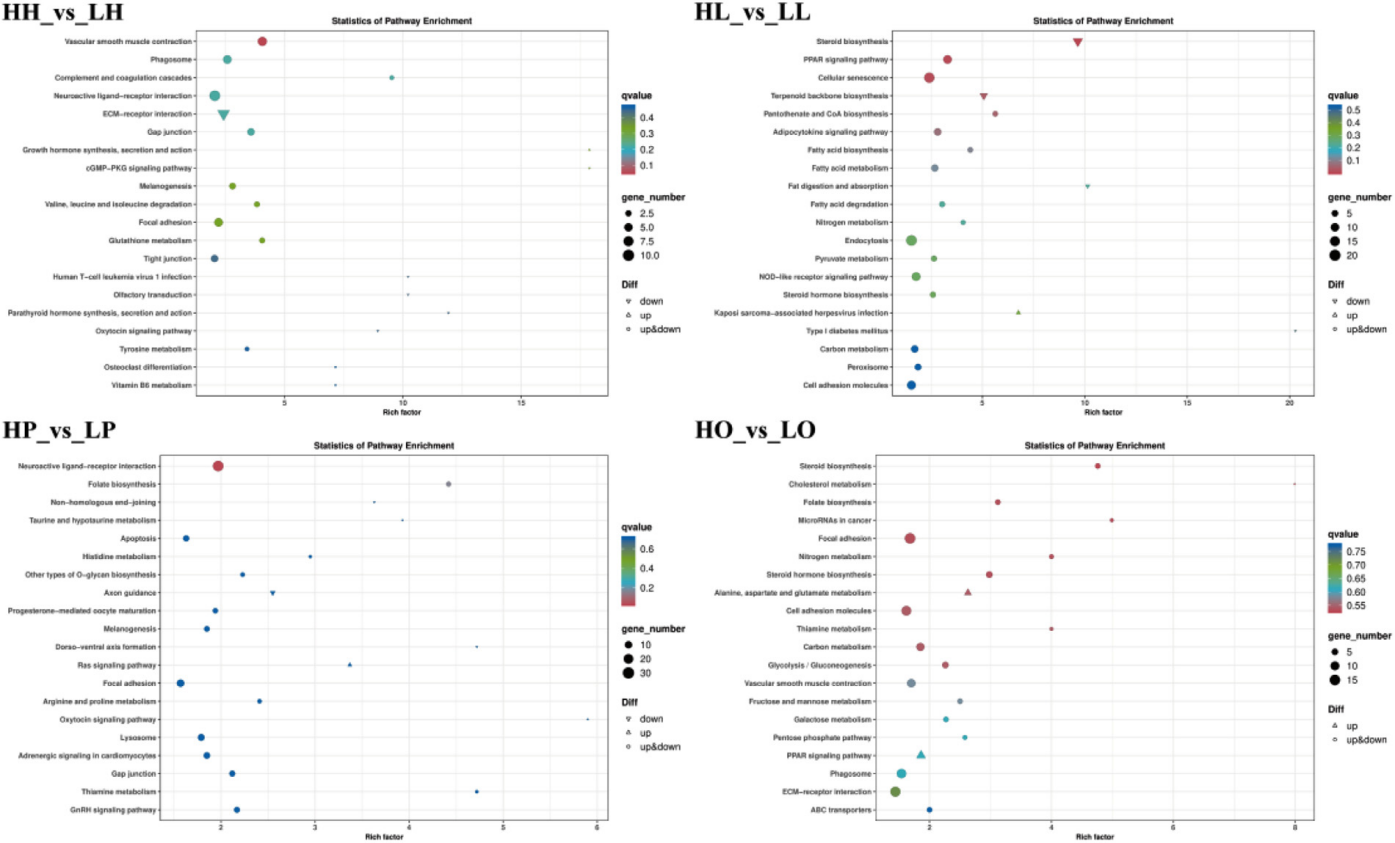
The top 20 significantly enriched KEGG terms in the 4 comparison pairs.

Pathway enrichment analysis identified the pathways significantly enriched by the DEGs. The KEGG pathways were significantly enriched by 77, 163, 171, and 170 DEGs from the HH_vs_LH, HP_vs_LP, HO_vs_LO, and HL_vs_LL groups, respectively (Supplementary Table S2 and Figure 4). The significantly enriched pathways in the hypothalamus and pituitary included “gap junction”, “extracellular matrix (**ECM**)-receptor interaction”, and “neuroactive ligand-receptor interaction”, all important pathways for signal transfer. Moreover, “steroid biosynthesis” and “steroid hormone biosynthesis”, important pathways of sex hormone synthesis, were significantly enriched in the liver and ovaries. Furthermore, other DEGs, including *PRL* and *CGA,* enriched "neuroactive ligand-receptor interaction" in the hypothalamic-pituitary axis (Supplementary Figure S2). However, *PRL* was significantly down-regulated in the LH and LP groups (*P* < 0.01), and *CGA* was significantly upregulated in both groups (*P* < 0.05).

### RNA Sequencing Validation by RT-qPCR

**Figure 5:**
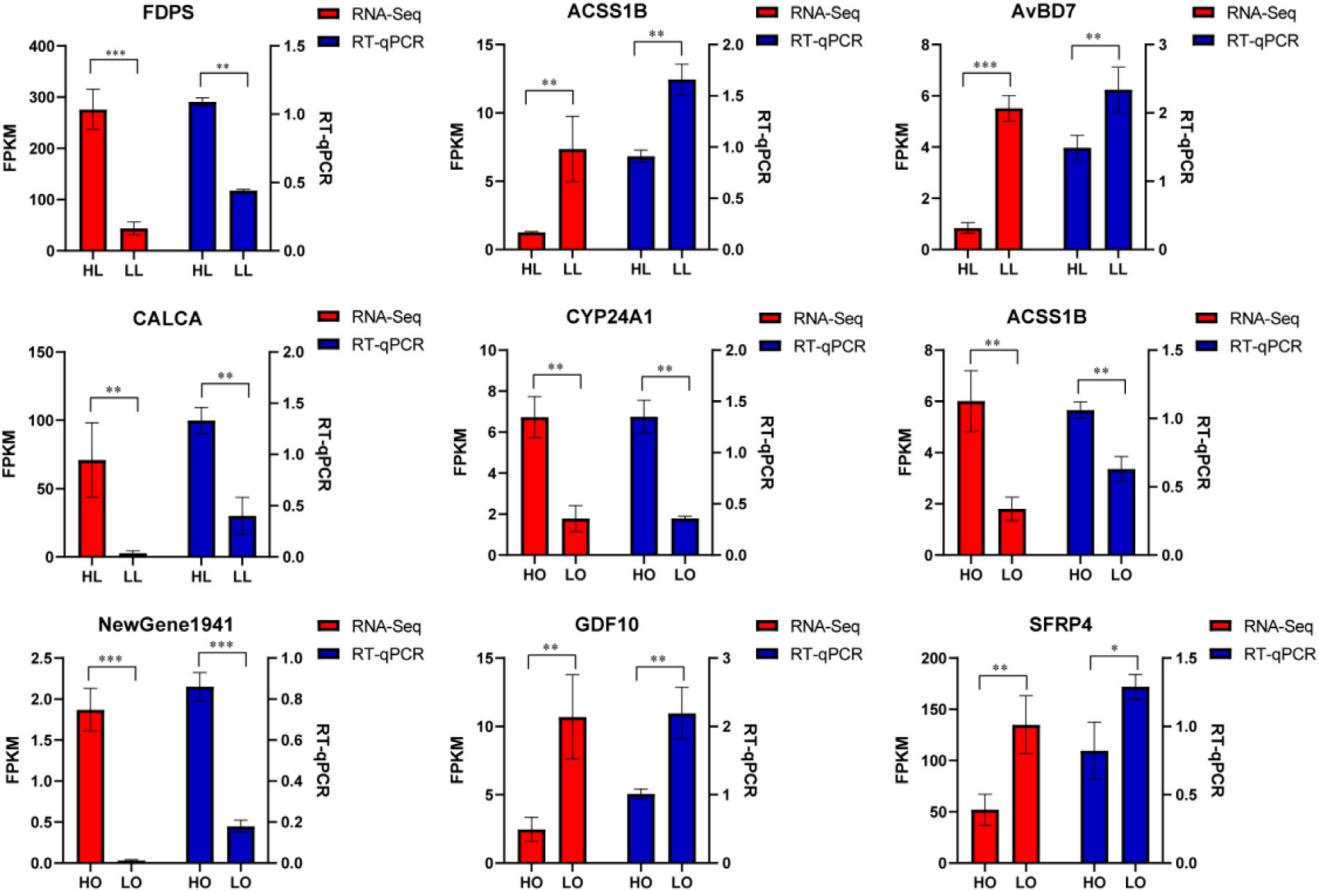
Validation of mRNA expression using RT-qPCR. The relative expression of 4 liver and 5 ovary mRNAs against ACTB mRNA.

Real-time, quantitative PCR (RT-qPCR) analysis validated the sequencing results. However, the validation was limited to the most significant differentially expressed mRNAs in the liver and ovaries (Figure 5), including *FDPS, ACSS1B, AvBD7, CALCA, CYP24A1, GDF10, SFRP4,* and *NewGene1941*. The RT-qPCR results were consistent with the RNA-seq.

### Metabolome Analysis of High- And Low-Laying Chickens

**Figure 6:**
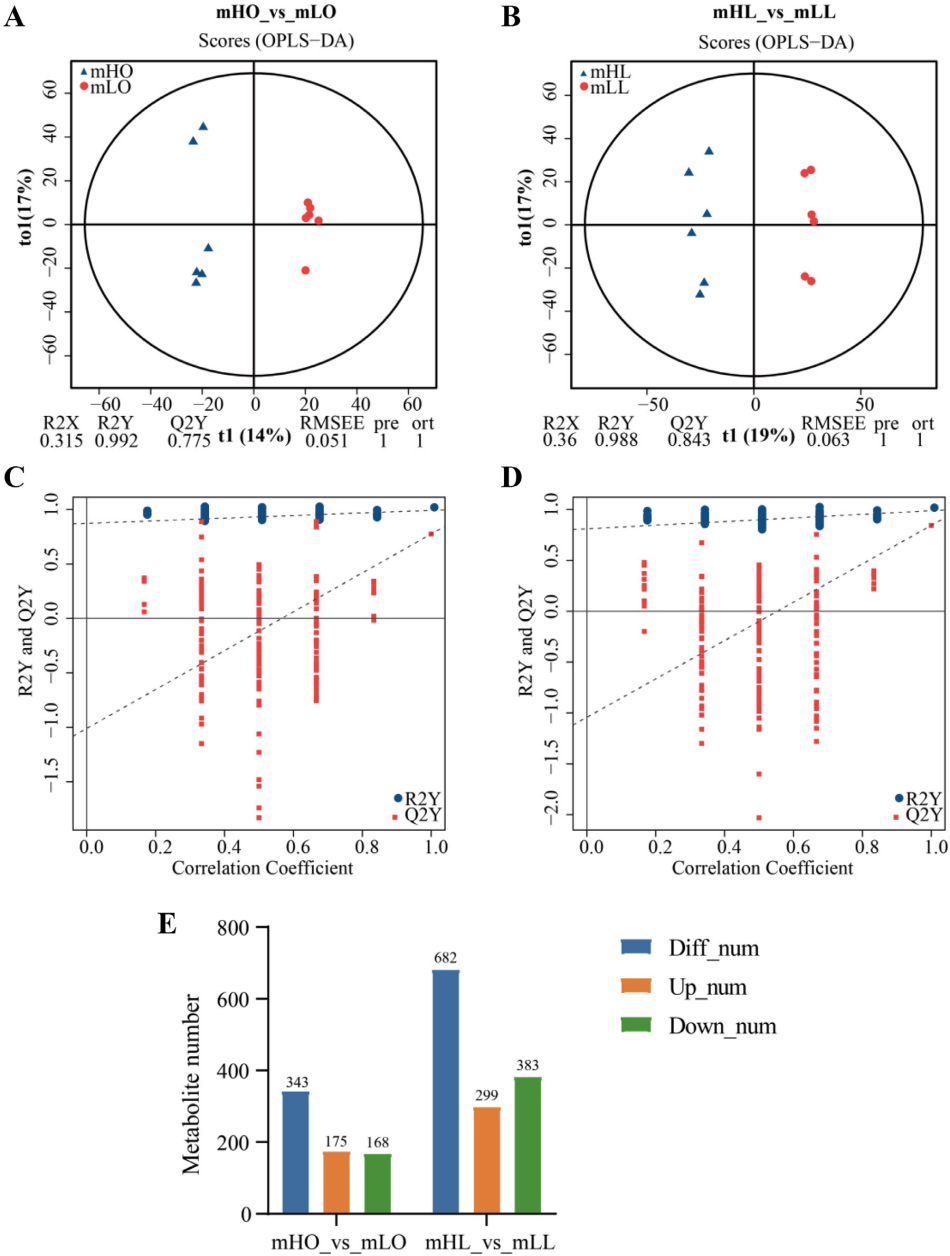
Metabolome data overview. (A) OPLS-DA model score plots for the group of mHO_vs_mLO**. (**B) OPLS-DA model score plots for the group of mHL_vs_mLL**.** (C) OPLS-DA validation for the group of mHO_vs_mLO**.** (D) OPLS-DA validation for the group of mHL_vs_mLL**.** (E) Statistic of SDMs.

Next, we considered whether the differential abundance of these mRNAs in different tissues was reflected in differences at the metabolite level. A total of 21,808 valid peaks were extracted via LC-MS/MS corresponding to 4,006 metabolites. Supplementary Figure S3 shows the reproducibility and dimensionality reduction PCA of the liver and ovary metabolome samples. An OPLS-DA elucidated the different metabolic patterns to maximize discrimination between the 2 groups. After 200 permutations, the R2Y value exceeded the Q2Y value, indicating a good modeling effect (Figures 6A–D).

Moreover, the low Q2Y intercept values indicate the robustness of the models, presenting low overfitting and reliability risks. All samples in the score plots were within the 95% Hotelling's T-squared ellipse, with clear separation and discrimination between the pairwise groups. The OPLS-DA could distinguish between the samples, thus rendering the model suitable for subsequent analysis.

**Figure 7:**
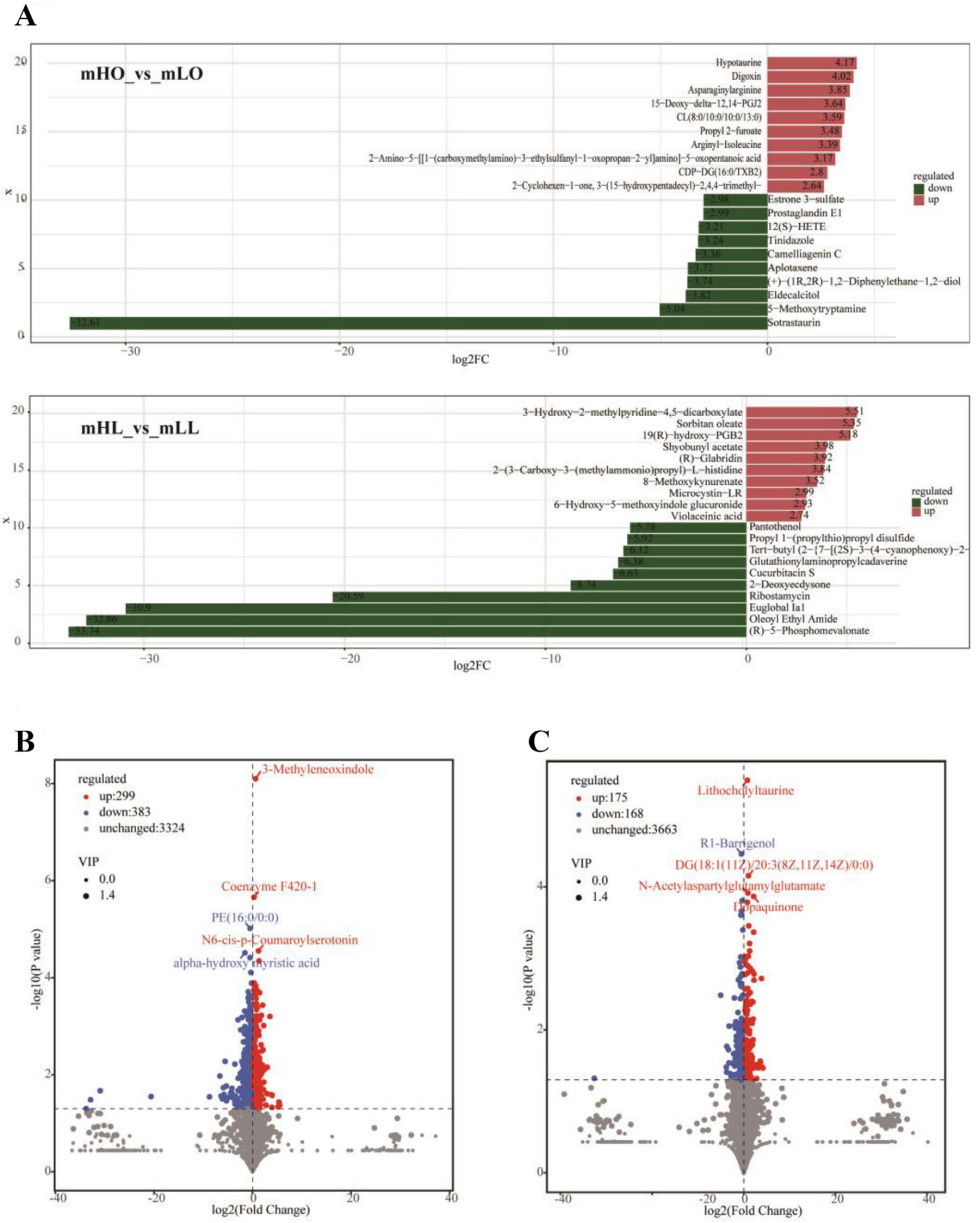
Differential expression of the metabolites in the liver and ovary of the high- and low-laying groups. (A) The log2FC of the top 20 significantly different metabolites (SDM) in the liver and ovary. (B, C) Volcano maps of the SDM in the liver and ovary.

The OPLS-DA results revealed 4,006 metabolites, including 343 significantly different metabolites (**SDMs**) and 682 SDMs in the mHL_vs_mLL and mHO_vs_mLO groups, respectively (Figure 6E and Supplementary Table S3). Moreover, the logFC of the top 10 metabolites upregulated and downregulated in each group were > 2. The logFC for mHO_vs_mLO (“sotrastaurin”) and those for mHL_vs_mLL (“Euglobal la1”, “Oleoyl Ethyl Amide”, “[R]-5-Phosphomevalonate”) exceeded 30 (Figure 7A). Meanwhile, the volcano map showed the top 5 SDMs with the lowest *P*-value. In the liver, the top 5 SDMs included 3 significantly upregulated metabolites (“3-Methyleneoxindole”, “Coenzyme F420-1″, and “N6-cis-p-Coumaroylserotonin”) and 2 significantly downregulated metabolites (“PE [16:0/0:0]” and “alpha-hydroxy mynstic acid”) (Figure 7B). Among the top 5 ovarian SDMs, 4 SDMs were significantly upregulated and “R1-Barrigenol” was significantly down-regulated (Figure 7C).

**Figure 8:**
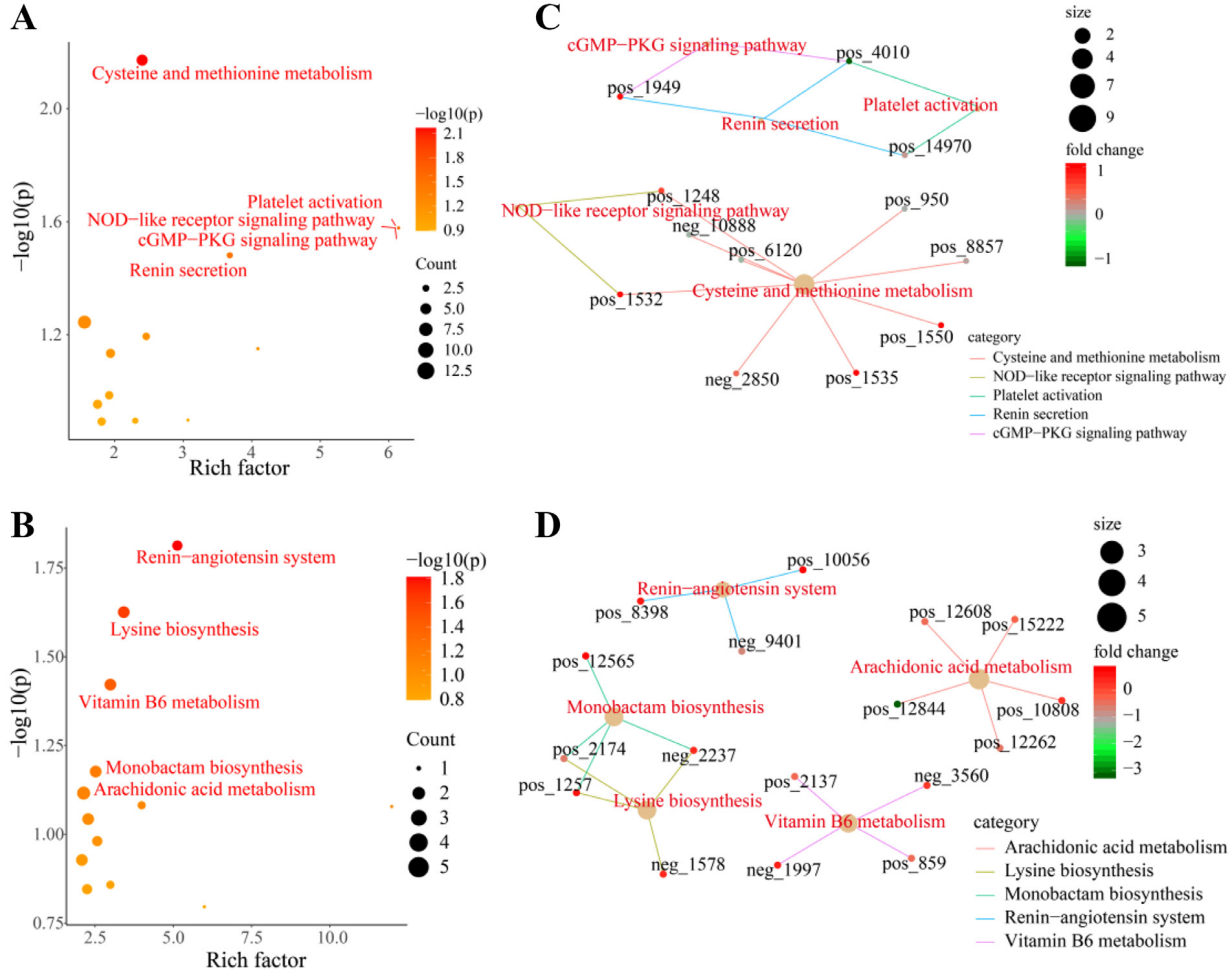
Relevant metabolic pathways in the liver and ovary. (A, B). Significantly enriched pathways in the liver and ovary. (C, D). Significantly enriched pathways and associated metabolites in the liver and ovary.

The SDMs enriched 136 (liver) and 87 (ovary) KEGG pathways (Supplementary Table S4). The -ln (*P*-value) and rich factor identified the most relevant metabolic pathways in the liver and ovaries as “cysteine and methionine metabolism”, “renin secretion”, “platelet activation”, “NOD-like receptor signaling pathway”, and “cGMP-PKG signaling pathway”, “renin-angiotensin system”, “lysine biosynthesis”, “vitamin B6 metabolism”, “monobactam biosynthesis”, and “arachidonic acid metabolism”, respectively (Figures 8A-B). Figures 8C-D shows the network between these pathways and their related metabolites.

### Integrative Analysis of Metabolome and Transcriptome

The transcriptome and metabolome data were analyzed using WGCNA dimensionality reduction, dividing the genes and metabolites into 32 metabolome and 124 transcriptome modules. The obtained heat maps (Supplementary Figure S4) showed strong correlations between each transcriptome and metabolome module, further highlighting the association of each gene set with specific metabolite sets.

**Figure 9:**
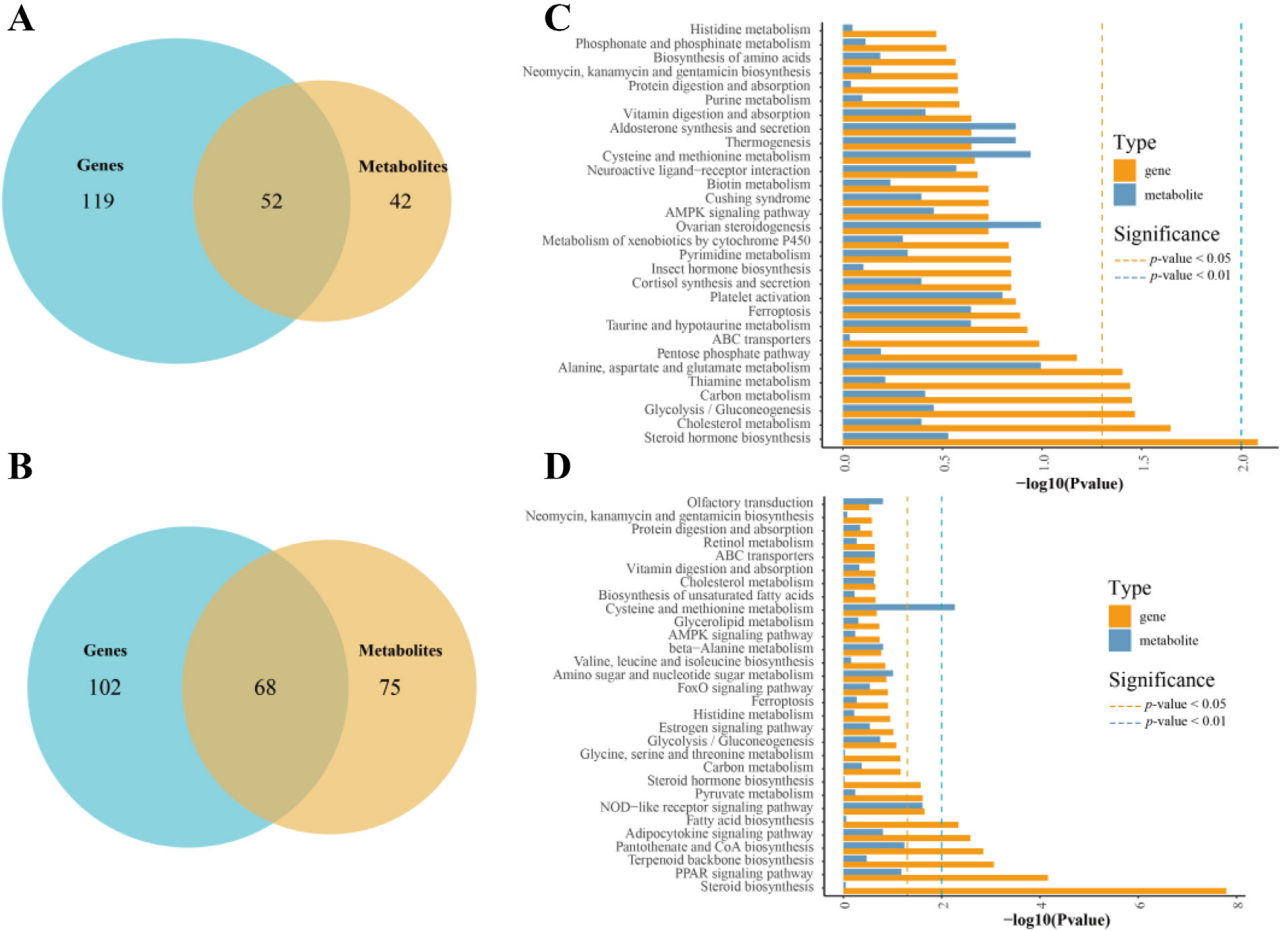
KEGG annotation of DEGs and SDM. (A, B). Venn diagram of enriched pathways and SDMs in the liver and ovary. (C, D). The top 30 pathways co-enriched by DEG and SDM. The orange dashed line represents *P* < 0.05, and the blue dashed line represents *P* < 0.01.

The KEGG annotation of genes and metabolites revealed the commonly enriched pathways, where the DEGs and SDMs in the HO_vs_LO group were enriched to 171 and 94 pathways, respectively. There were 52 pathways present in both results, 119 pathways were enriched in the DEGs, and 42 pathways were enriched in the SDMs (Figure 9A). Similarly, DEGs and SDMs in the HL_vs_LL group were enriched to 170 and 143 pathways, respectively. There were 68 pathways present in both results, 102 pathways were enriched in the DEGs and 75 pathways were enriched in the SDMs (Figure 9B). The top 30 significantly enriched pathways in the liver included “steroid biosynthesis”, “PPAR signaling pathway”, “terpenoid backbone biosynthesis”, “pantothenate and CoA biosynthesis”, “adipocytokine signaling pathway”, “fatty acid biosynthesis”, “NOD-like receptor signaling pathway”, “pyruvate metabolism”, “steroid hormone biosynthesis”, and “cysteine and methionine metabolism”. Those in the ovaries included “steroid hormone biosynthesis”, “cholesterol metabolism”, “glycolysis/gluconeogenesis”, “carbon metabolism”, “thiamine metabolism”, and “alanine, aspartate, and glutamate metabolism”. Additionally, the SDMs in the ovaries significantly enriched several nonsignificant pathways, including “ovarian steroidogenesis”, “cysteine and methionine metabolism”, “thermogenesis” and “aldosterone synthesis and secretion” (Figures 9C-D).

**Figure 10:**
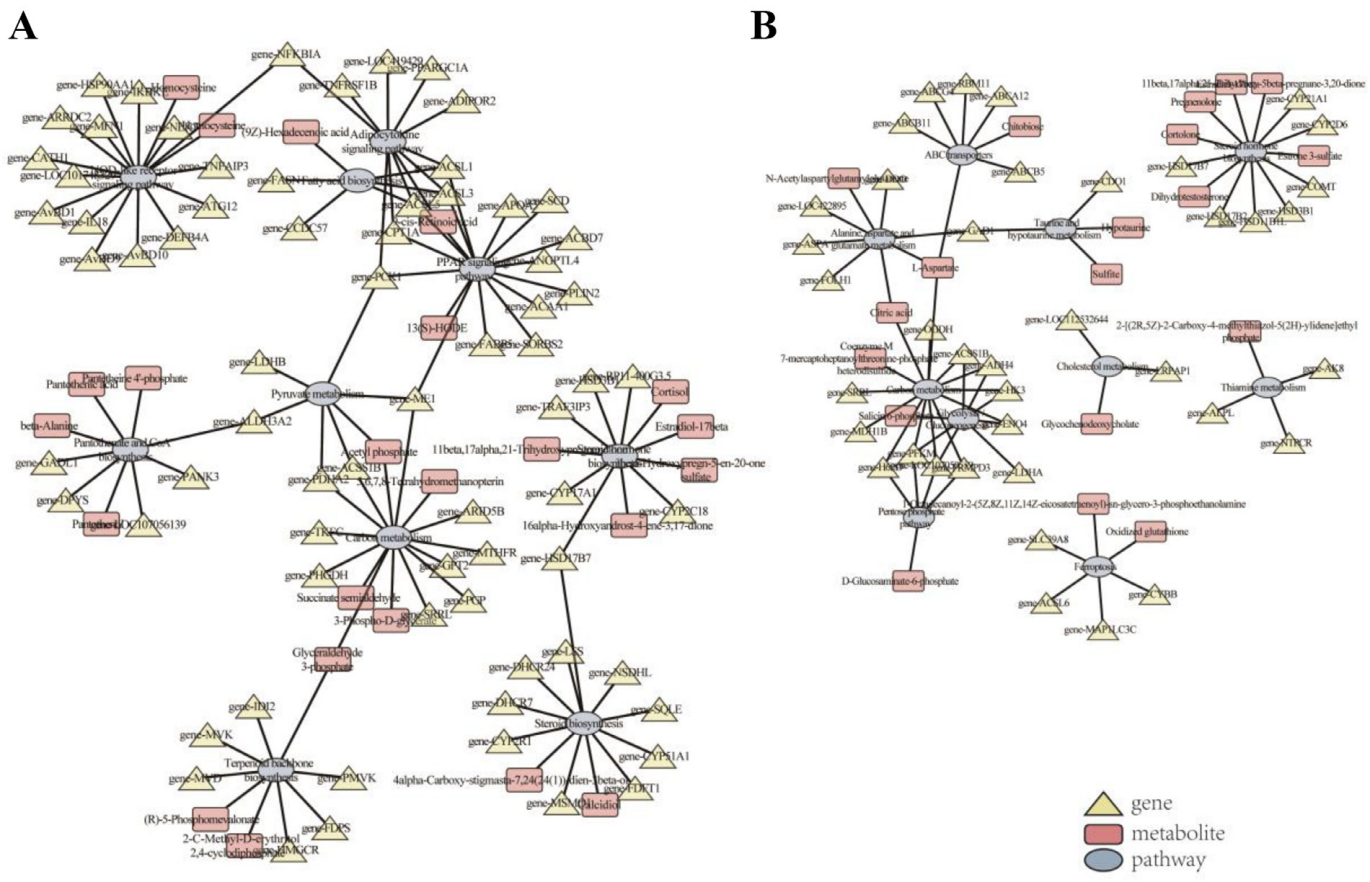
KGML network analysis. (A) Liver. (B) Ovary.

In the liver, DEGs, SDMs, and pathways were mainly divided into 2 regulatory networks: 1 steroid-related and 1 signal transduction and substance metabolism (Figure 10A, Supplementary Table S5). In the ovary, 5 networks were enriched, but energy metabolism and amino acid metabolism networks were the largest, while steroid hormone biosynthesis was the most significant (Figure 10B, Supplementary Table S5). In the steroid biosynthesis pathway, “calcidiol” and “4α-carboxy-stigmasta-7,24(24[1])-dien-3β-ol” were significantly downregulated in the LL compared to the HL group (Supplementary Figure S6A). Pearson's correlation analysis revealed that “4α-carboxy-stigmasta-7,24 (24[1])-dien-3β-ol” and “calcidiol” were positively and significantly correlated with *AvBD9/10, CCDC57, CYP51A1, LSS*, and *NSDHL* (*P* < 0.01), and negatively and significantly correlated with *ACSS1B, AvBD1, CATH1, DEFB4A*, and *FABP5* (*P* < 0.01, Supplementary Figure S5A). In steroid hormone biosynthesis, the levels of “pregnenolone” were significantly increased and the levels of “11β,17α,21-Trihydroxy-5β-pregnane-3,20-dione”, “estrone 3-sulfate”, “estradiol-17β”, “5α-dihydro-testosterone”, and “cortolone” were significantly downregulated in the LO compared to the HO group (Supplementary Figure S6B). Likewise, “estrone 3-sulfate” was positively and significantly correlated with *ABCB5, ACSS1B*, and *SLC39A8* (*P* < 0.01), while “pregnenolone” and “cortolone” were negatively and significantly correlated with *H6PD, CYP21A1*, and *HSD11B1L* (*P* < 0.01, Fig. S5b).

## DISCUSSION

Eggs are an important protein resource for humans, and improving egg production is an important goal for the poultry industry. The laying performances of local chicken breeds differ considerably from those of commercial laying hens (Du et al., 2020), providing a large space for development and utilization. The ovary is an important reproductive organ in poultry, and ovarian health and normal development are crucial for egg production (Xiang et al., 2023). Furthermore, the liver is the major organ for energy and fatty acid metabolism (Pearce, 1971). Yolk deposition in the follicle depends on the transport of lipids and proteins from the liver to the ovary (Lv et al., 2018). The hypothalamic-pituitary axis also affects the reproductive performance of animals by regulating the liver and ovaries. Therefore, this study investigated the morphology and histology of the liver and ovaries in high- and low-laying groups under the same external conditions.

The high-laying group had a yellowish liver color, a more ordered hepatocyte arrangement, and uniformly distributed lipid droplets. This result is inconsistent with those of Tang (Tang et al., 2023), probably due to differences in the chicken breed and laying period. Some studies have shown that oil red O staining in the liver of poultry with early fatty livers can produce red precipitates and white vacuoles (Liu et al., 2024, Y et al., 2023, Liu et al., 2022a). Our study found that oil red O staining in low-laying hens also showed lipid precipitates, suggesting that the low egg production of laying hens may as a result of the use of energy in liver fat deposition. The ovarian hierarchical follicles in the high-laying group were rich in yolk and granulosa cells consistent with the findings of Cui (Cui et al., 2022), which may be the basis for high-laying hens.

The RNA-seq data from the hypothalamus, pituitary, liver, and ovary contained 285, 822, 787, and 1,183 DEGs, respectively. The DEGs in the hypothalamus and pituitary significantly enriched these pathways: “neuroactive ligand-receptor interaction”, “ECM-receptor interaction”, “gap junction”, and “folate biosynthesis”. The first 3 are related to signal transmission and “folate biosynthesis” is bound up with folic acid synthesis. Thus, the hypothalamus and pituitary, important components of the nervous and endocrine systems, are highly active in and conducive to maintaining the normal life activities of organisms (Chen et al., 2017, Adamantidis and de Lecea, 2023). Interestingly, *TFPI2, FKBP5, HNMT, TPH2, ALPL*, and *MCHR2* were differentially expressed in the hypothalamus and pituitary, important organs in the neuroendocrine system.

Studies on *TFPI2* have mainly focused on glioblastoma, which is related to neurodevelopment (Pang et al., 2023). However, *TFPI2* can inhibit the expression of *NPVF* and regulate the expression of the synaptic excitation-related gene, *FKBP5* (Matosin et al., 2023), thereby promoting the secretion of *GnRH* (Wang, 2023) and *FKBP5* through the *PI3K/AKT* and NF-κB signaling pathways. These pathways regulate the recurrent, spontaneous abortion trophoblastic-macrophage crosstalk, indicating that *FKBP5* is crucial in maintaining pregnancy and the maternal-fetal interface trophoblastic macrophage crosstalk (Chen et al., 2023). In this study, *TFPI2* and *FKBP5* were down-regulated in group LH, suggesting they may play important roles in regulating egg production traits.

Histamine N-methyltransferase (***HNMT***) is a histamine-metabolizing enzyme expressed in the brain to regulate neural activity by metabolizing histamine (Haas et al., 2008, Yoshikawa et al., 2019). Brain histamine is a neurotransmitter and regulates diverse physiological functions. Serotonin is another important neurotransmitter in diseases such as gastrointestinal disease (Gershon and Tack, 2007), cognitive control (Waider et al., 2011), and emotional regulation (Kulikova and Kulikov, 2019). Serotonin is synthesized by 2 different tryptophan hydroxylases (*TPH1/2*), where *TPH2* is mainly expressed in the brain, while *TPH1* is mainly expressed in peripheral tissues such as the gut (Bader, 2020, Jones et al., 2020). Both enzymes (*TPH1/2*) were significantly down-regulated in low-laying chickens, implying that when the chickens ceased to lay eggs, the body entered the rehabilitation period, decreasing the brain, nerve and gastrointestinal tract activities.

The *ALPL* (Alkaline Phosphatase, Biomineralization Associated) mutations cause hypophosphatemia, mainly characterized by defects in bone and tooth mineralization (Mohamed et al., 2022). The significantly higher expression of *ALPL* in low-laying chickens may indicate that the chickens are using more nutrients for bone repair. Melanin-concentrating hormone (***MCH***) signaling has a broad endocrine background and is implicated in physiological functions and emotional states related to metabolism, such as reproduction, anxiety, depression, sleep, and circadian rhythms (Al-Massadi et al., 2021). The decreased expression of *MCHR2* (Melanin Concentrating Hormone Receptor 2) indicated that the energy metabolism of chickens decreased with the reduction of egg production. Also, *MCHR2*, as the receptor of *MCH*, is involved in feeding behavior and energy metabolism (An et al., 2001).

In both the liver and ovaries, “steroid biosynthesis” and “steroid hormone biosynthesis” were significantly enriched, while fatty acid-related pathways were enriched in the liver, and “folate biosynthesis” was enriched in the ovaries. In the liver, “steroid biosynthesis” was significantly inhibited, whereas “steroid hormone biosynthesis” was significantly activated. Similarly, the liver and ovaries contained potential candidate genes (*TGFBR1, ADH4, ACSS1B, HK3, LDHB, ACAA1*, and multiple genes of the *CYP450* family and *HSD* family) for egg production. The *TGFBR1* (transforming growth factor beta receptor 1) gene is a key member of the TGF-β family and is critical in the TGF-β signaling pathway. It is associated with high fecundity in the Hu sheep (Li et al., 2010). In this study, the level of *TGFBR1* in liver tissues was higher in the HL than in the LL group. Thus, decreasing *TGFBR1* concentration induced nesting.

The *CYP* (Cytochrome P450) gene belongs to monooxygenase, involved in the metabolism of many substances, while *HSD* (Hydroxy-Delta-5-Steroid Dehydrogenase) is mainly involved in the biosynthesis of steroid hormones, such as testosterone, estradiol, and progesterone. Many studies have revealed that the *CYP* and *HSD* family genes are closely related to reproductive traits (Yao et al., 2021, Wu et al., 2023, Zhu et al., 2023, de Jong et al., 2014, Lu et al., 2023, Kordus et al., 2019). Yuan (Tang et al., 2023) observed significant differences in *ACSS1B, ADH4,* and *HK2* protein levels in the liver of local chicken and commercial chicken breeds that lay eggs after the late ovarian stage. The Yuan (Tang et al., 2023) results are consistent with this study, confirming the reliability of the transcriptome analysis.

The liver and ovarian metabolome contained 343 and 682 SDMs, respectively. The SDMs enriched “euglobal la1”, “Oleoyl Ethyl Amide”, “(R)-5-Phosphomevalonate” in the liver, and “sotrastaurin” in the ovary. “Sotrastaurin” is a protein kinase C inhibitor that blocks T cell activation, playing a role in cancer (Yuan et al., 2019, Bauer et al., 2022), bone disease (Pang et al., 2020), and early rejection in organ transplantation treatment (Pascher et al., 2015). Although they were not the most significant SDMs, the impact of “sotrastaurin” on poultry egg production is worth considering.

Additionally, bile acids, ADP, and estradiol concentrations were significantly higher in mHL/mHO than in mLL/mLO. Bile acid promotes the digestion and absorption of fat (Hu et al., 2019), a precursor of ATP. Besides, ADP is essential for energy regulation and estradiol, the major hormone that promotes follicular development is produced by follicular granulosa cells and controls the development and selection of dominant preovulatory follicles (Chauvin et al., 2022). Thus, ovarian atrophy in low-laying chickens decreased the estradiol content, decreasing the demand for fat.

In the ovary, the SDMs enriched numerous amino acid metabolism pathways. For instance, aspartic acid and citrate were significantly decreased in group mLO, affecting the TCA cycle, alanine metabolism, aspartate metabolism, and glutamate metabolism. Thus, decreasing egg production decreases the requirement for amino acids and energy. In group mLL, ovarian atrophy decreased the concentrations of metabolites such as hexadecenoic acid, 25-hydroxycholecalciferol, 4α-carboxy-stigmasta-7, 24 (24 [1]) -dien-3β-ol and cortisol. Positional isomers of hexadecenoic acid have anti-inflammatory effects (Astudillo et al., 2020), and hexadecenoic acid is an important component of egg fatty acid and flavor (Dong et al., 2021). Bonagurio (Bonagurio et al., 2022) showed that dietary supplementation of 25-hydroxycholecalciferol and canthaxanthin improved the reproductive performance of European quail.

Therefore, integrating the transcriptome and metabolome determined the potential gene-metabolite pairs regulating egg production in chickens. In the liver, the potential genes include *CYP51A1, LSS, ACSS1B,* and *FABP5*, and the metabolites were 4α-carboxy-stigmasta-7,24(24[1])-dien-3β-ol and calcidiol, all involved in various steroid biosynthesis processes. In the ovaries, *ABCB5, ACSS1B, SLC39A8, H6PD, CYP21A1*, and *HSD11B1L* were the potential genes, with estrone 3-sulfate, pregnenolone, and cortolone as candidate metabolites, all related to the steroid hormone biosynthetic pathway.

## CONCLUSION

This study showed that the DEGs in the hypothalamus and pituitary are mainly involved in nerve signal transduction, while the DEGs and SDMs in the liver and ovaries are mainly involved in carbohydrate, lipid, and amino acid metabolism. These results lay a theoretical foundation for further developing and utilizing high-quality local chicken breeds in China, enriching the research in the poultry community and providing a reference value for related scientific research.

## METHODS

### Animals and Sample Collection

The Animal Committee of Southwest University reviewed and approved all the poultry experiments (No. LAC2024-1-0083). In the preliminary experiment, we counted the laying of chickens from the beginning of laying to the age of 400 d and found that CMC maintained a high egg production rate up to 350 d of age. From 300 to 350 d of age is the stage of hatching egg collection for CMC, and 350 d of age was therefore selected as the follow-up sampling time point. The egg production of chickens at 350 d was counted (Supplementary Figure S7), and the results were consistent with the normal distribution trend. Therefore, the 20% range at both ends was set as the sampling interval, the chickens with egg production in the left sampling interval were set as the low-laying group, and the chickens with egg production in the right sampling interval were set as the high-laying group. Based on this, we randomly selected 3 chickens from each group, the chickens were of the same weight (2,407.5 g ± 11.09 g), in good health, and were managed under the same conditions at the Chengkou Mountain Chicken Research Institute. The chickens were fed the same amount of feed twice a day at 08:00 a.m. and 18:00 p.m. and drank freely. The chickens were sacrificed by cervical dislocation after ether respiratory anesthesia, and hypothalamus (group HH/LH, HH: high-laying chicken hypothalamus, LH: low-laying chicken hypothalamus), pituitary (group HP/LP), liver (group HL/LL), and ovarian (group HO/LO, the yolks from hierarchical follicles were eliminated) samples were collected from each chicken and immediately frozen in liquid nitrogen for transport to the laboratory. The ovarian samples were further processed in the laboratory, and the whole ovarian sample was fully ground and mixed under liquid nitrogen, divided into aliquots, and cryopreserved at −80°C. Total RNA was extracted from 1 liver, ovary, hypothalamus, and pituitary sample for transcriptome sequencing. The second sample of liver (group mHL/mLL) and ovary (group mHO/mLO) were used for metabolome sequencing.

### Hematoxylin–Eosin and Oil Red O Staining

The samples were first immobilized in 4% paraformaldehyde (Solarbio, Beijing, China) for 24 h, and then immersed in 70, 80, 90, 95, and 100% ethanol solutions for 30 min each to dehydrate the samples. Next, the samples were bleached in xylene for 2 h before embedding in paraffin wax for 3 h. The embedded samples were sectioned into 5 μm pieces and immersed in xylene for 20 min to dewax before immersion in a series of ethanol solutions from high to low concentrations and finally in distilled water. The sections were stained with a hematoxylin solution (Beyotime, Haimen, China) for 4 min, fractionated in hydrochloric acid and ethanol for 3 s each, rinsed in running water for 1 h, immersed in distilled water for 10 min, and dehydrated in 70% and 90% ethanol for 10 min each, followed by eosin staining for 3 min. The stained sections were dehydrated by immersion in an ethanol solution, then immersion in xylene to make the sections transparent before finally sealing and staining with gum.

Oil red O staining was performed following the manufacturer's protocol. Briefly, samples were washed in a phosphate-buffered saline (**PBS**), fixed with the ORO Fixative for 30 min, and the stationary fluid was discarded before washing twice in PBS. Thereafter, 60% isopropanol was added to the sample and maintained for 5 min, then ORO Stain was added and maintained for 20 min. Furthermore, Mayer Hematoxylin staining solution was added to the samples and incubated for 2 min before washing 5 times. Another washing followed before adding ORO Buffer to the samples and sustaining for 1 min. The samples were washed with PBS and covered with distilled water. All sections and samples were viewed under a DP80Digital electronic microscope (Olympus, Tokyo, Japan).

### cDNA Library Construction and Sequencing

Total RNA was extracted using TRlzol Reagent (Life Technologies, CA) following the manufacturer's instructions. The RNA concentration and purity were measured using a NanoDrop 2000 (Thermo Fisher Scientific, DE). Then, RNA integrity was assessed using the RNA Nano 6000 Assay Kit of the Agilent Bioanalyzer 2100 system (Agilent Technologies, CA). Yeasen Biotechnology (Shanghai Co., Ltd., Shanghai, China) generated the sequencing libraries using the Hieff NGS Ultima Dual-mode mRNA Library Prep Kit for Illumina (Illumina, CA) following the manufacturer's recommendations. Briefly, mRNA was purified from total RNA using poly-T oligo-attached magnetic beads. First strand cDNA was synthesized followed by second strand cDNA synthesis. The remaining overhangs were converted into blunt ends via exonuclease/polymerase activity and after adenylation of the 3′ ends of the DNA fragments, a NEBNext Adaptor with a hairpin loop structure was ligated to prepare for hybridization. The library fragments were purified with the AMPure XP system (Beckman Coulter, Beverly, USA) and 3 μl of USER Enzyme (NEB, USA) was added to the size-selected, adaptor-ligated cDNA at 37°C for 15 min, followed by 5 min at 95°C before PCR. The PCR was performed with Phusion High-Fidelity DNA polymerase, Universal PCR primers and an Index (X) Primer. At last, PCR products were purified with the (AMPure XP system) and library quality was assessed on the Agilent Bioanalyzer 2100 system (Agilent Technologies, CA). The libraries were sequenced on an Illumina NovaSeq platform (Illumina, CA) to generate 150 bp paired-end reads, according to the manufacturer's instructions.

### Transcriptome Analysis

The raw reads were processed using the online bioinformatic pipeline tool, BMKCloud (www.biocloud.net) platform. Clean reads were obtained by removing reads containing adapter sequences, ploy-Ns, and low-quality reads from the raw data. The Q20, Q30, GC-content, and sequence duplication levels were further used to retain clean data for subsequent analysis. All downstream analyses were based on clean, high-quality data. The clean reads were then mapped to the reference genome sequence using Hisat2 (Kim et al., 2015). Only perfectly matched reads or those with 1 mismatch was further annotated to the reference genome. StringTie (Pertea et al., 2015) was used to construct and identify both known and novel transcripts from the Hisat2 alignment. Differential expression analysis was performed using DESeq2 (Love et al., 2014). Genes with FDR < 0.01 and |log2(FC)| ≥ 1 (FC: Fold Change) were considered DEGs. A hypergeometric test using the KEGG database (Kanehisa et al., 2004) identified the significantly enriched pathways against the entire genome background.

### Metabolites Extraction and LC-MS/MS Analysis

Metabolite extraction and LC-MS/MS analysis were performed as described previously (Cui et al., 2022). Briefly, 50 mg of samples were placed in 1.5 mL centrifuge tubes. Acetonitrile, methanol, and water at a ratio of 2:2:1 were added to the internal standard (2-chloro-L-phenylalanine). Next, 1 mL of the miscible liquids was placed in a centrifuge tube, mixed thoroughly, homogenized at 45 Hz for 10 min, and subsequently sonicated for 10 min (ice water bath). Then, the samples were incubated at −20°C for 1 h and centrifugation for 15 min (12,000 rpm, 4°C). The supernatants were carefully absorbed (500 μL) in EP tubes and dried in a vacuum concentrator. Then, 160 μL extract solution (acetonitrile and water at a ratio of 1:1) was added to each dried metabolite sample for re-dissolution and mixed well for 30 s. The mixtures were incubated in an ice water bath ultrasound for 10 minutes and centrifuged for 15 minutes (12,000 rpm, 4°C). Next, 120 μL of each supernatant was placed in a 2 mL sample vial. Simultaneously, 10 μL of each sample was mixed with the QC sample for machine testing.

The LC−MS/MS analysis was performed using the Acquity I-Class PLUS ultra-high-performance liquid (Waters Corp, MA) with a Xevo G2-XS QTOF high-resolution mass spectrometer (Waters Corp, MA) and a UPLC HSS T3 column (2.1 mm × 100 mm, 1.8 μm). The mobile phase conditions of mobile phase A was 0.1% formic acid (CAS: 64-18-6) aqueous solution, and for mobile phase B it was 0.1% formic acid acetonitrile (positive mode same as negative mode). The mass spectrometry parameters were as follows: positive ion mode ESI voltage 2,500 V, negative ion mode − 2,000 V, cone hole voltage: 30 V, ion source temperature: 100°C, de solvent gas temperature: 500°C, desolventizing gas flow rate: 800 L/h, and backflush gas flow rate: 50 L/h, karyoplasmic ratio (m/z) collection range 50-1200. Finally, the primary and secondary mass spectrometry data were collected using a Xevo G2-XS QTOF high-resolution mass spectrometer (Waters Corp, MA).

The raw data was collected using MassLynx V4.2 (Waters Corp, MA) and processed with the Progenesis QI software (Waters Corp, MA, USA). Peak extraction, peak alignment, and other data processing operations were based on the Progenesis QI software online METLIN database and a self-built library for identification (Biomark, ID). All theoretical fragment identification and mass deviations were within 100 ppm. The original peak area information was normalized with the total peak area before the follow-up analysis. Principal component analysis and Spearman correlation determined the repeatability of the samples within a group and the quality control samples. The identified compounds were classified using the pathway information in the KEGG, HMDB (Wishart et al., 2018), and LIPID MAPS (Fahy et al., 2007) databases. The grouping information was used to calculate and compare the difference multiples, and T-tests determined the significance of the *p*-values of each compound. The R language package “ropls” was used to perform OPLS-DA modeling with 200 permutations to verify the reliability of the model. The variable important in projection (**VIP**) model values was calculated using multiple cross-validation. Finally, the difference multiple, *P*-value and VIP value of the OPLS-DA model were combined to identify the significantly different metabolites (**SDM**). The screening criteria were FC > 1, *P*-value < 0.05, and VIP > 1. The hypergeometric distribution test calculated the SDMs that significantly enriched the KEGG pathway.

### Integrative Analysis of the Metabolome and Transcriptome

The DEGs (FDR < 0.01 and |log2FC| ≥ 1) and SDMs (FC > 1, VIP > 1, and *P* < 0.05) were used for integrative analysis of the high- and low-laying groups. First, the correlation between gene and metabolite modules was determined using the R package “WGCNA”. Next, the pathway model was used to analyze the significantly enriched KEGG metabolic pathways. The DEGs and SDMs associated with common metabolic pathways were visualized using Cytoscape (V3.9.1). The relationship between DEGs and SDMs was determined using Pearson's correlation.

### RT-qPCR Validation

The samples extracted for RNA-seq were used for quantitative reverse transcription polymerase chain reaction (RT-qPCR). Primer Premier v. 5.0 software (Premier Biosoft International, CA) was used to design the primers (Table S6), and RT-qPCR was performed as previously described (Ren et al., 2021), with *ACTB* as the housekeeping gene. The RT-qPCR procedure was performed in the CFX96 PCR instrument (Bio-Rad, CA) using the TB Green Premix Ex Taq (Tli RNaseH Plus) kit (TaKaRa Bio Inc., Shiga, Japan) following the manufacturer's instructions. Relative expression was calculated using the 2^-ΔΔCT^ method and each assay was repeated 3 times.

### Statistical Analysis

All results are presented as mean ± standard error of the mean (SEM). One-way ANOVA was conducted using SPSS 26.0 (IBM Corporation, NY), and LSD was used for the post hoc test to determine the difference between means at the *P < 0.05, **P < 0.01, and ***P < 0.001 significance levels.

## DISCLOSURES

Authors have no conflict of interest to declare.

## Appendix Supplementary materials

Image, application 1

Image, image 2

Image, image 3

Image, image 4

Image, image 5

Image, image 6

Image, image 7

Image, image 8

Image, application 9

Image, application 10

Image, application 11

Image, application 12

Image, application 13

Image, application 14

## Footnotes

Supplementary material associated with this article can be found in the online version at doi:10.1016/j.psj.2024.104276.
