# Supplementary material for: Multiomics analyses reveal high yield-related genes in the hypothalamic-pituitary-ovarian/liver axis of chicken
Source: Poult Sci. 2024 Sep 2;103(12):104276. doi: 10.1016/j.psj.2024.104276 (PMC11426133; doi:10.1016/j.psj.2024.104276)
Supplement: Supplementary file 1 [file mmc1.docx]

**Supplementary Materials**

Supplementary Table 1. Sequencing data statistics.

Supplementary Table 2. Statistical of alignment results between sequencing data and reference genomes.

Supplementary Table S1. Table of differentially expressed genes.

Supplementary Table S2. Pathways enrichment results of differentially expressed genes.

Supplementary Table S3. Table of significantly different metabolites.

Supplementary Table S4. Pathways enrichment results of significantly different metabolites.

Supplementary Table S5. KGML network node information.

Supplementary Table S6. Table of RT-qPCR primers. F: Forward primer, R: Reverse primer.

**Supplementary Figure S1. Results of the genomic alignment of all samples.**

**Supplementary Figure S2. Differentially expressed genes in neuroactive ligand-receptor interaction.** Red and dark green color represent upregulated and downregulated, respectively.

**Supplementary Figure S3. Overview of the metabolomic data. a.** Heatmap of sample correlation, darker red indicates greater correlation, and darker green indicates less correlation. **b.** Principal component analysis (PCA) distribution of all samples, ellipses indicate 95% confidence intervals.

**Supplementary Figure S4. Heatmap of module correlation for WGCNA analysis of transcriptome data and metabolome data.** Red and green represent positive and negative correlations, respectively, with darker colors being more correlated. *** means *p* < 0.001, ** means *p* < 0.01, * means *p* < 0.05.

**Supplementary Figure S5. Heatmap of differentially expressed genes and significantly differential metabolites correlation in kgml analysis. a.** Liver. **b.** Ovary. Red and blue represent positive and negative correlations, respectively, with darker colors being more correlated. ** means *p* < 0.01, * means *p* < 0.05.

**Supplementary Figure S6. Differentially expressed genes and metabolites in pathways. a.** Steroid biosynthesis. **b.** Steroid hormone biosynthesis. Red and green color represent upregulated and downregulated, respectively. Rectangles and circles represent genes and metabolites, respectively.

**Supplementary Figure S7. Egg production statistics**.
