# Supplementary figures and images for: Multiomics analyses reveal high yield-related genes in the hypothalamic-pituitary-ovarian/liver axis of chicken
Source: Poult Sci. 2024 Sep 2;103(12):104276. doi: 10.1016/j.psj.2024.104276 (PMC11426133; doi:10.1016/j.psj.2024.104276)

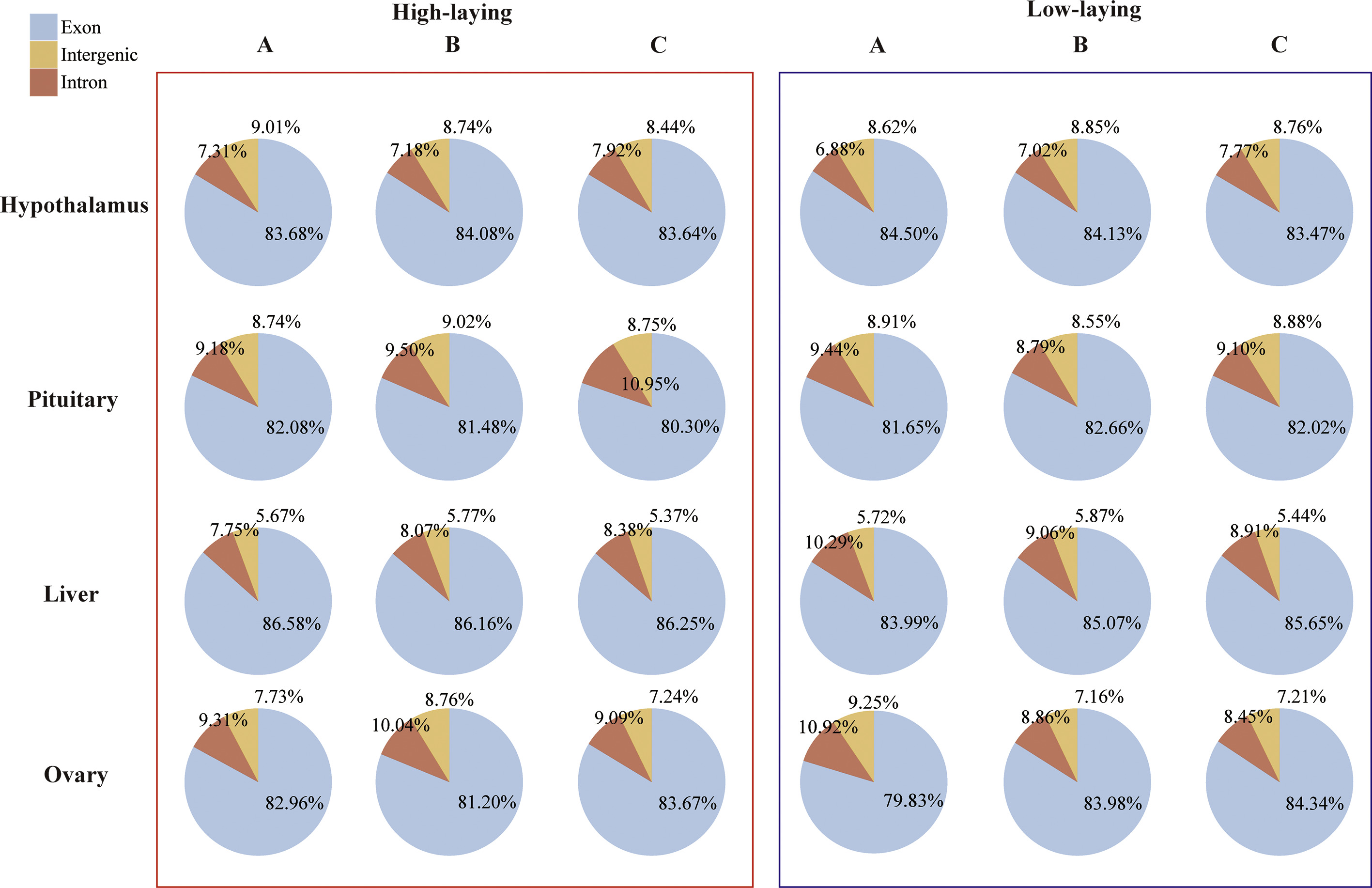

Supplement: Supplementary file 2 [file mmc2.jpg]

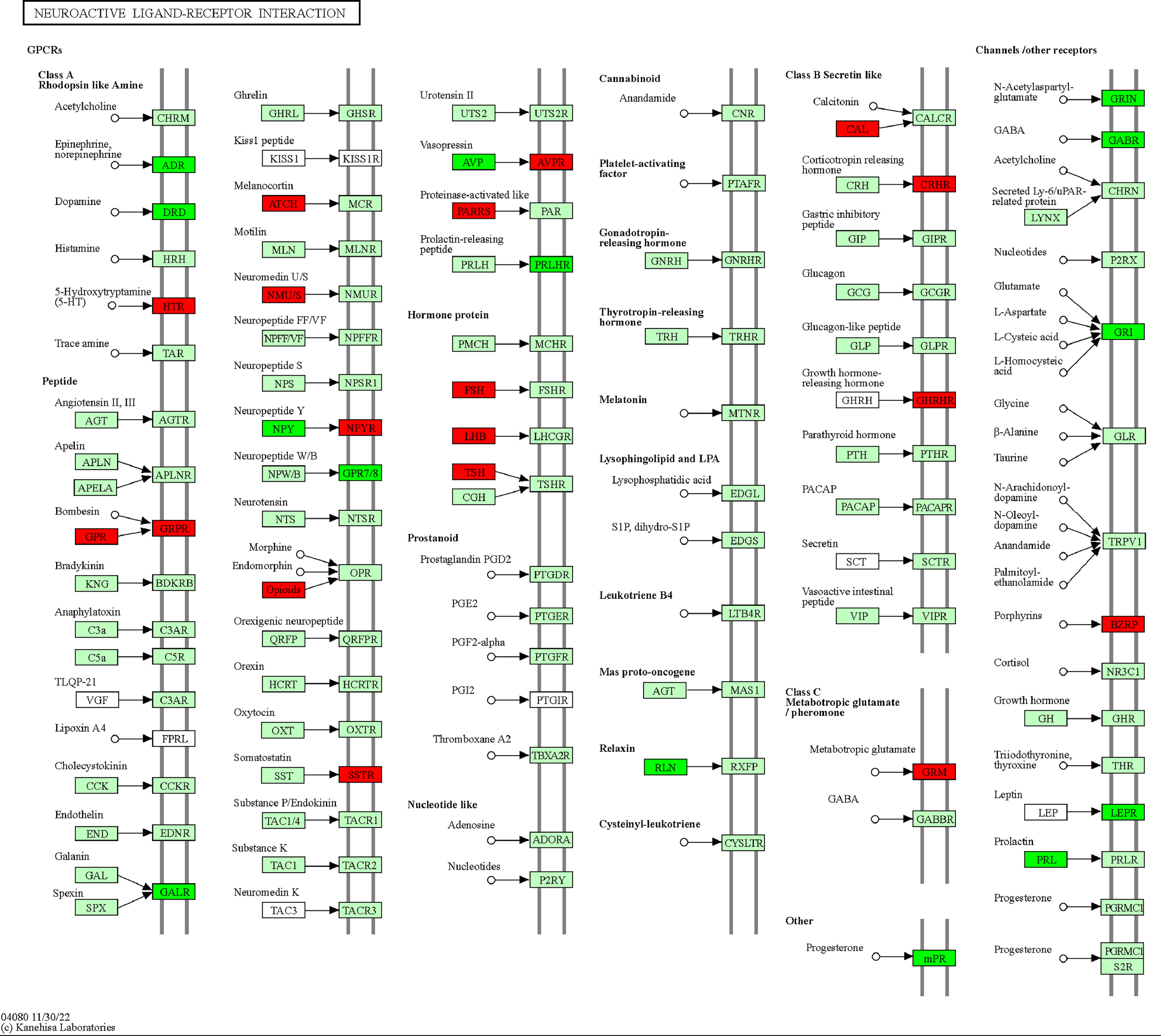

Supplement: Supplementary file 3 [file mmc3.jpg]

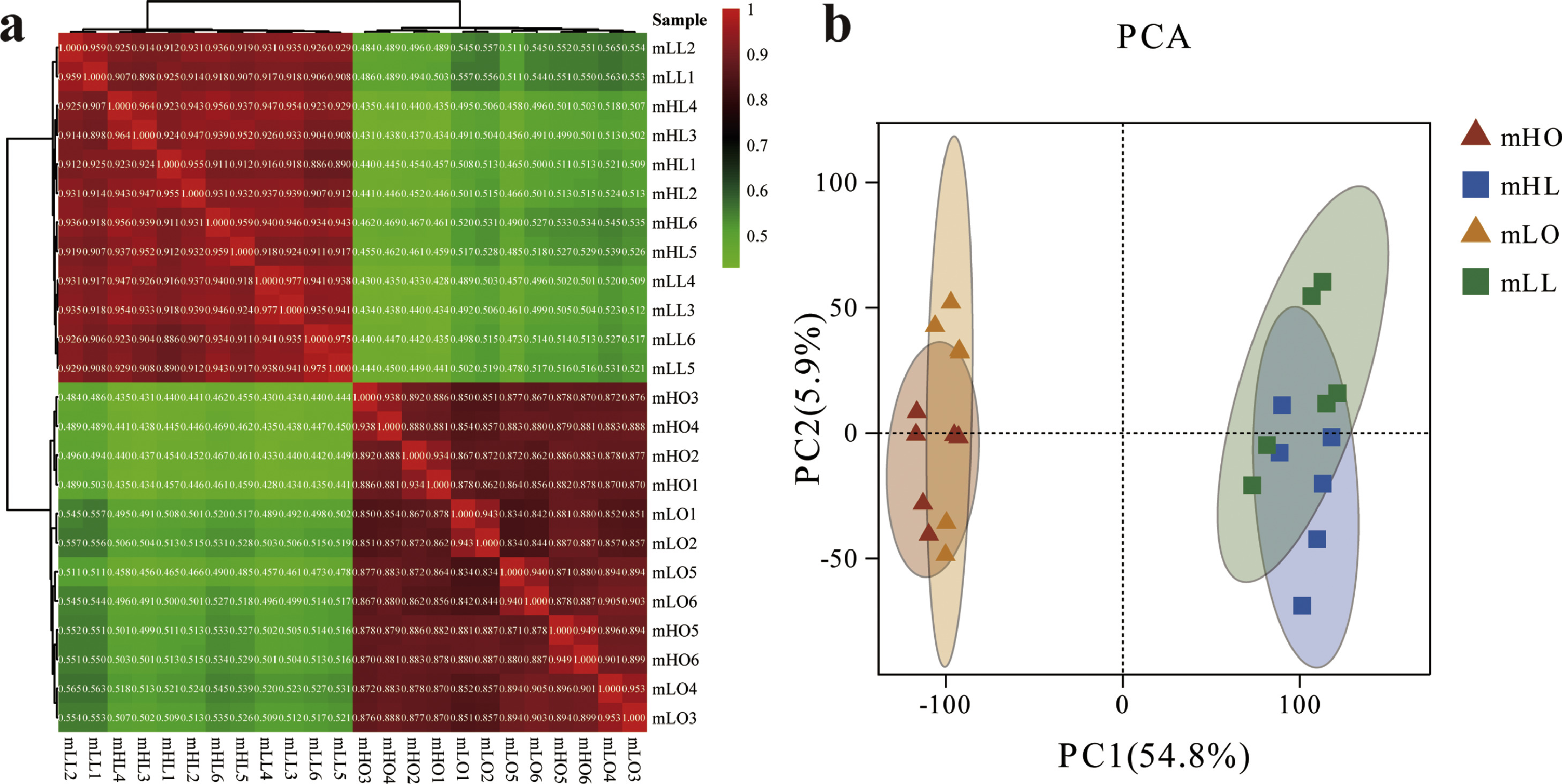

Supplement: Supplementary file 4 [file mmc4.jpg]

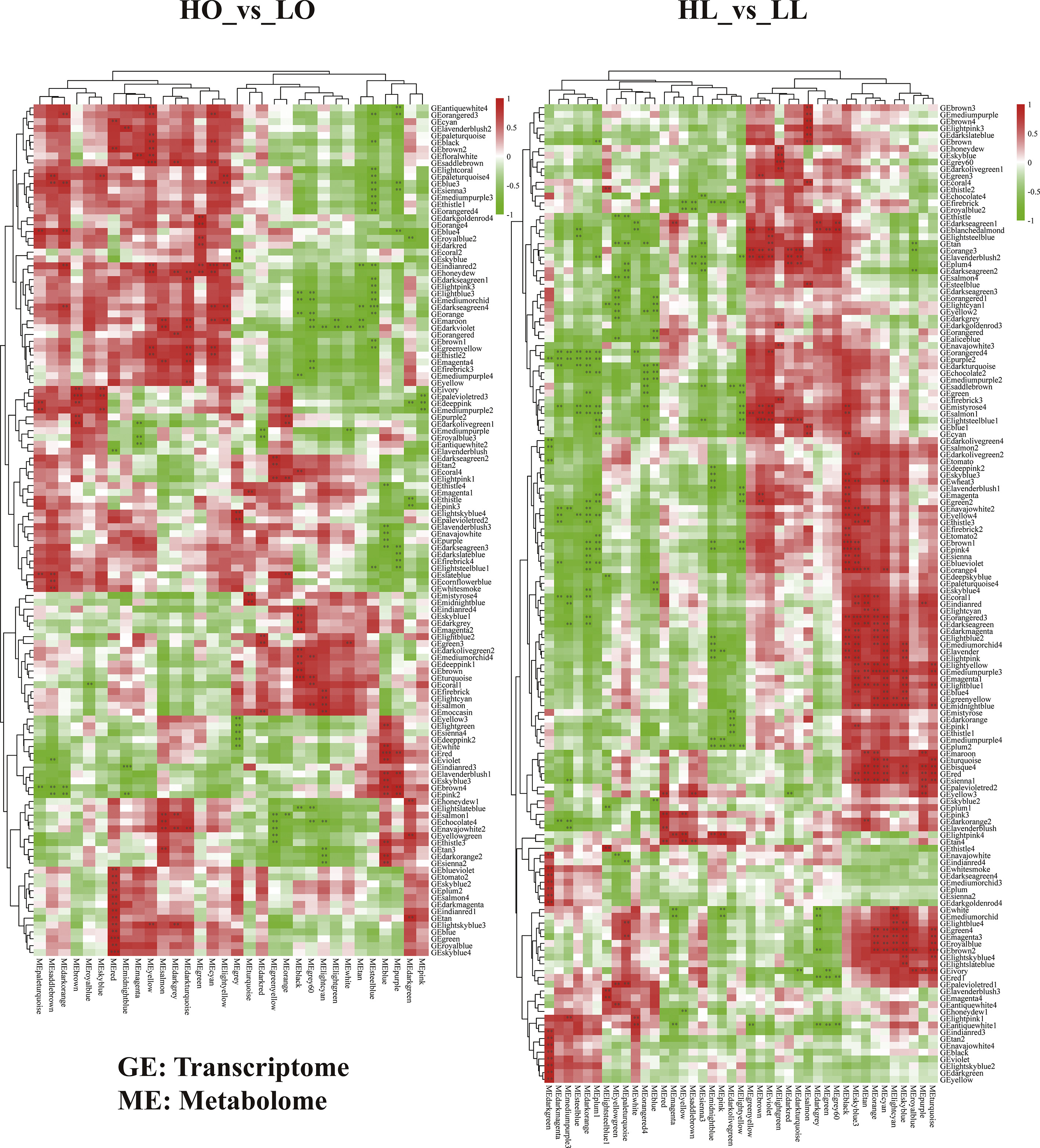

Supplement: Supplementary file 5 [file mmc5.jpg]

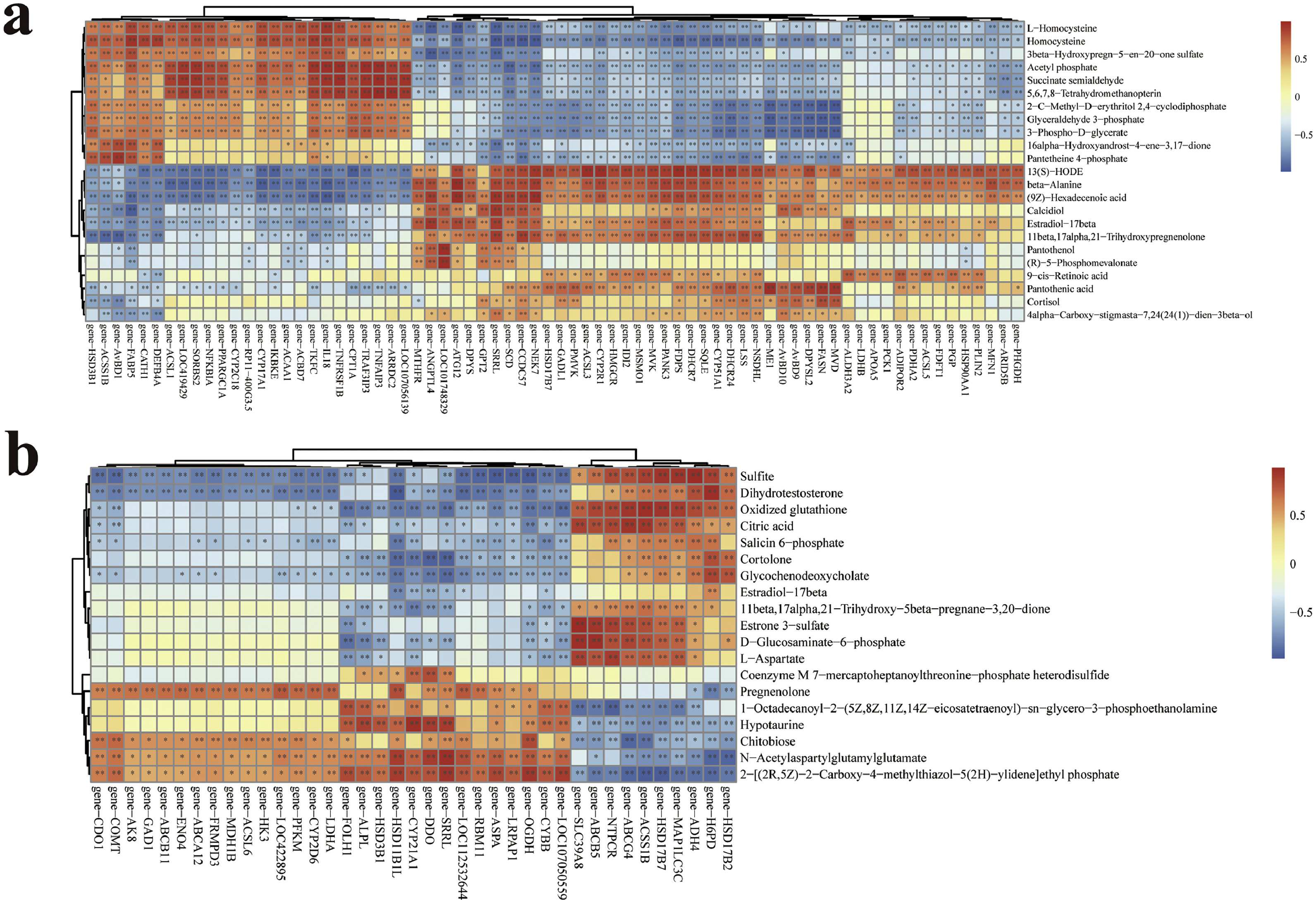

Supplement: Supplementary file 6 [file mmc6.jpg]

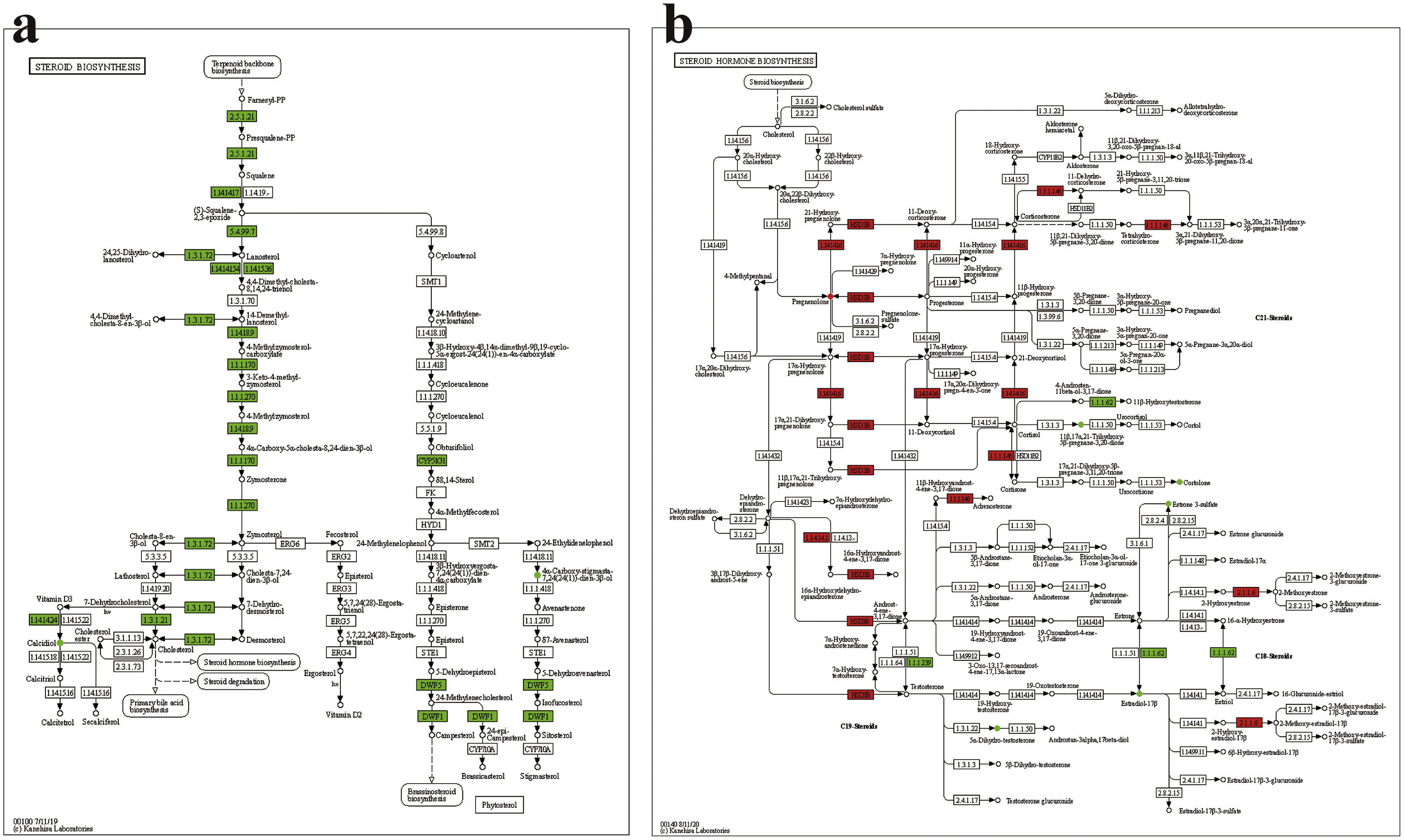

Supplement: Supplementary file 7 [file mmc7.jpg]

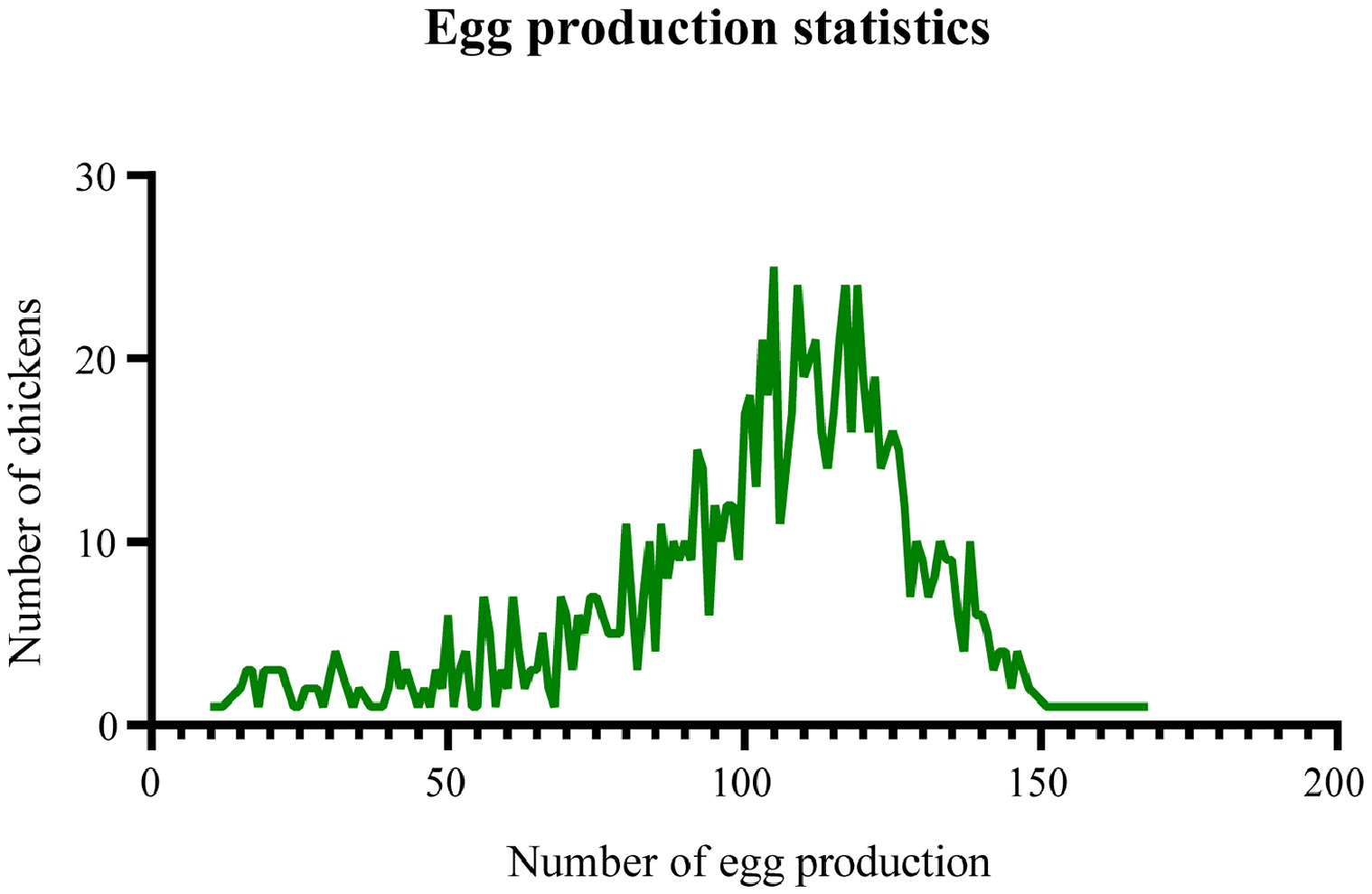

Supplement: Supplementary file 8 [file mmc8.jpg]
